# Mapping the Regulatory Network for *Salmonella enterica* Serovar Typhimurium Invasion

**DOI:** 10.1128/mBio.01024-16

**Published:** 2016-09-06

**Authors:** Carol Smith, Anne M. Stringer, Chunhong Mao, Michael J. Palumbo, Joseph T. Wade

**Affiliations:** a Wadsworth Center, New York State Department of Health, Albany, New York, USA; b Biocomplexity Institute of Virginia Tech, Virginia Tech, Blacksburg, Virginia, USA; c Department of Biomedical Sciences, School of Public Health, University at Albany, Albany, New York, USA

## Abstract

*Salmonella enterica* pathogenicity island 1 (SPI-1) encodes proteins required for invasion of gut epithelial cells. The timing of invasion is tightly controlled by a complex regulatory network. The transcription factor (TF) HilD is the master regulator of this process and senses environmental signals associated with invasion. HilD activates transcription of genes within and outside SPI-1, including six other TFs. Thus, the transcriptional program associated with host cell invasion is controlled by at least 7 TFs. However, very few of the regulatory targets are known for these TFs, and the extent of the regulatory network is unclear. In this study, we used complementary genomic approaches to map the direct regulatory targets of all 7 TFs. Our data reveal a highly complex and interconnected network that includes many previously undescribed regulatory targets. Moreover, the network extends well beyond the 7 TFs, due to the inclusion of many additional TFs and noncoding RNAs. By comparing gene expression profiles of regulatory targets for the 7 TFs, we identified many uncharacterized genes that are likely to play direct roles in invasion. We also uncovered cross talk between SPI-1 regulation and other regulatory pathways, which, in turn, identified gene clusters that likely share related functions. Our data are freely available through an intuitive online browser and represent a valuable resource for the bacterial research community.

## IMPORTANCE

Invasion of epithelial cells is an early step during infection by *Salmonella enterica* and requires secretion of specific proteins into host cells via a type III secretion system (T3SS). Most T3SS-associated proteins required for invasion are encoded in a horizontally acquired genomic locus known as *Salmonella* pathogenicity island 1 (SPI-1). Multiple regulators respond to environmental signals to ensure appropriate timing of SPI-1 gene expression. In particular, there are seven transcription regulators that are known to be involved in coordinating expression of SPI-1 genes. We have used complementary genome-scale approaches to map the gene targets of these seven regulators. Our data reveal a highly complex and interconnected regulatory network that includes many previously undescribed target genes. Moreover, our data functionally implicate many uncharacterized genes in the invasion process and reveal cross talk between SPI-1 regulation and other regulatory pathways. All datasets are freely available through an intuitive online browser.

## INTRODUCTION

*Salmonella enterica* is the causative agent of typhoid fever and is also a major cause of foodborne illness (salmonellosis) (1). There are many serovars of *S. enterica* that cause salmonellosis; one of the clinically most important serovars is *S. enterica* subsp. *enterica* serovar Typhimurium (2). During the initial stages of infection, *S*. Typhimurium invades gut epithelial cells. The invasion process requires injection of specific effector proteins into host cells through a type III secretion system (T3SS). This T3SS and most of the secreted effector proteins are encoded in *Salmonella* pathogenicity island 1 (SPI-1), a horizontally acquired chromosomal region of ~40 kbp (3). Under standard laboratory growth conditions, SPI-1 genes are transcriptionally repressed. However, during the initial stages of infection, SPI-1 genes are induced in response to environmental triggers. These triggers can be mimicked in the laboratory by growth in media containing high levels of salt, by low levels of aeration, and by growth to the late exponential/early stationary phase (3–6). The master regulator of SPI-1 genes is HilD, an AraC family transcription factor (TF) encoded within SPI-1 (7, 8). HilD expression and activity are controlled by multiple pathways that sense the environmental cues associated with invasion (8, 9). HilD activates transcription of several SPI-1 genes, including components of the T3SS, secreted effector proteins (10–12), and the TFs HilA and InvF (7, 10, 11). HilA and InvF activate transcription of additional T3SS components and effector proteins (8, 13–16). Approximately half of the known HilD-regulated genes are located outside SPI-1 (11). Notably, HilD activates transcription of genes within *Salmonella* pathogenicity island 4 (SPI-4), which are required for attachment of *S*. Typhimurium to epithelial cells (11, 17). HilD also activates transcription of genes whose function has not been directly connected to invasion, such as *lpxR*, which encodes an enzyme that modifies lipid A (11), and *flhDC*, which encodes the master regulator of flagellar motility (11, 18, 19). Regulation of genes not involved in invasion may be a mechanism to coordinate their expression with the invasion process (11) or may indicate an as-yet-unidentified role in the invasion process.

In addition to HilD, HilA, and InvF, four other TFs within SPI-1, or connected to SPI-1, have been associated with host cell invasion. HilC and RtsA are close homologues of HilD, and their regulons overlap that of HilD (7, 8, 10, 11, 20, 21). HilC is encoded within SPI-1, whereas RtsA is encoded elsewhere in the genome. HilD, HilC, and RtsA not only regulate shared target genes but also activate each other’s transcription (8, 10, 11, 20). Thus, these proteins represent a positive-feedback loop for activation of SPI-1 genes (20, 22). SprB is a TF encoded within SPI-1 also; however, little is known about the contribution of SprB to global gene regulation (23). Transcription of *sprB* is induced under conditions associated with invasion (4, 23), although the mechanism of this regulation is unknown. Only one regulatory target has been described for SprB: the operon that encompasses SPI-4 (24). RtsB is a TF encoded outside SPI-1 but connected to SPI-1 due to its regulation by HilD/HilC/RtsA (10). Moreover, RtsB is encoded in an operon with RtsA. The only known regulatory target of RtsB is *flhDC* (10, 19). However, unlike HilD, RtsB is a negative regulator of *flhDC* (10, 19). Thus, flagellar motility is regulated both positively and negatively by SPI-1-associated TFs (19). Although the precise functions of the 7 TFs described above have not been described, for simplicity we refer to them as “SPI-1-associated TFs.”

Levels of active HilD, HilC, RtsA, and HilA are controlled by many different regulators, indirectly impacting expression of other SPI-1 genes (9). Nucleoid-associated proteins (NAPs) are abundant DNA-binding proteins that bind large numbers of genomic locations, typically with little sequence specificity (25). Several NAPs play crucial roles in regulating SPI-1 gene expression. For example, H-NS binds extensively within SPI-1 (26, 27), leading to repression of many SPI-1 genes, including *hilD*, *hilC*, *rtsA*, and *hilA* (28–31). Two other NAPs, Fis and HU, positively regulate SPI-1 genes, although the mechanism of regulation is unknown and may be indirect (32–35). Direct, positive regulation of SPI-1 genes is often due to displacement of H-NS by other DNA-binding proteins (“countersilencing” [36]). Indeed, this is the case for activation of *hilD*, *hilC*, *rtsA*, and *hilA* by HilD/HilC/RtsA (29, 30). Another NAP, IHF, also positively regulates *hilA* by displacing H-NS (31). In contrast, several transcription factors positively or negatively regulate SPI-1 genes by controlling *hilD* transcription independently of H-NS (e.g., Fur) (37, 38), or by controlling HilD translation (e.g., SirA and CsrA) (39) or activity (e.g., HilE and FliZ) (40, 41). Transcription factors also control SPI-1 gene expression by regulating *hilC* (OmpR) (35, 42) or *hilA* (PhoP and FNR) (5, 9, 43). By controlling expression/activity of HilD, HilC, RtsA, and HilA, many regulators contribute to SPI-1 gene regulation in response to environmental stimuli that include temperature (H-NS and Hha), osmolarity (H-NS and Hha), pH (OmpR), iron availability (Fur), and oxygen levels (FNR).

HilD and HilA targets have been identified on a genomic scale using chromatin immunoprecipitation (ChIP) methods and transcription profiling (11, 13). However, some of the regulatory targets for HilA that have been identified using genome-scale approaches are inconsistent with targeted investigations of individual transcripts (18, 24). Moreover, there have been no genome-scale investigations of the regulons of HilC, RtsA, RtsB, InvF, or SprB. Hence, the extent of SPI-1-associated gene regulation is largely unknown. Here, we used a combination of ChIP sequencing (ChIP-seq) and transcriptome sequencing (RNA-seq) to comprehensively map the direct regulatory targets of SPI-1-associated TFs under conditions that mimic the host environment during the invasion process. Thus, we have generated a critical resource for understanding the regulation associated with epithelial cell invasion. Combining ChIP-seq and RNA-seq data allows us to identify all regulated genes and to distinguish direct and indirect regulatory targets. All ChIP-seq and RNA-seq data are freely available for viewing using an intuitive genome browser (http://www.wadsworth.org/research/scientific-resources/interactive-genomics or http://salmonella.wadsworth.org). Our data reveal a complex web of regulation, with all SPI-1-associated TFs being regulated by at least one other. We also identified many novel regulatory targets for SPI-1-associated TFs. Expression of these genes is therefore tightly coordinated with the invasion process. Notably, many of the novel regulatory targets encode TFs or small RNAs (sRNAs), indicating that the regulatory network associated with invasion extends well beyond the known SPI-1-associated TFs. By comparing gene expression profiles for the regulatory targets of the seven analyzed TFs, we identified 17 genes whose expression closely mirrors that of known SPI-1 genes. These genes are therefore likely to play direct roles in invasion. We also identified clusters of genes with similar expression profiles, suggesting cross talk between invasion gene regulation and other regulatory pathways.

## RESULTS AND DISCUSSION

### Identification of direct regulatory targets for HilD.

**FIG 1:**
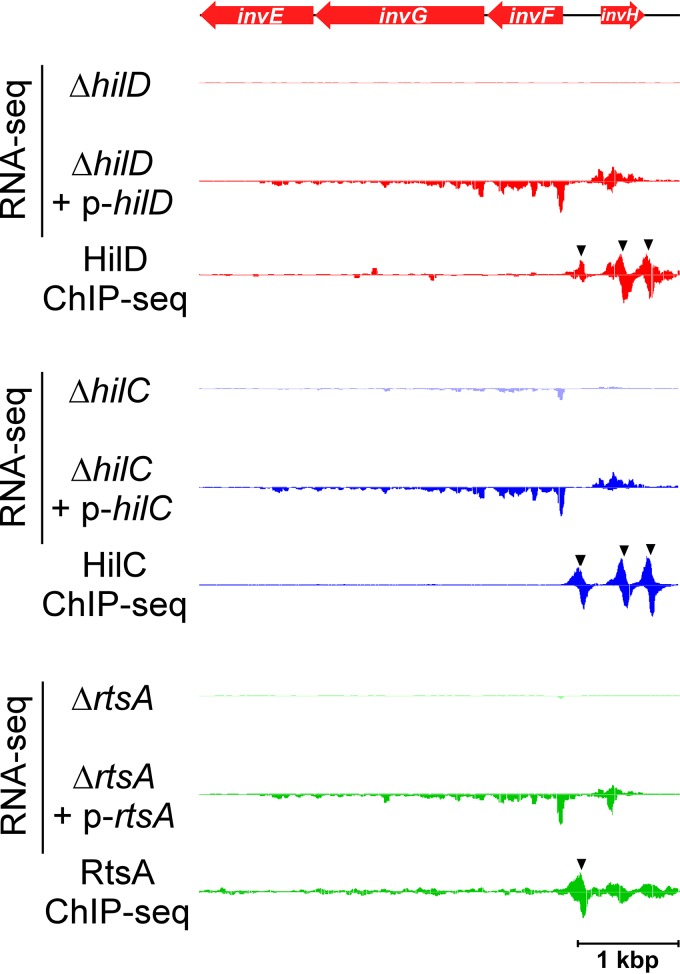
Direct regulation of the *invF*-containing transcript by HilD, HilC, and RtsA. Data represent results of RNA-seq and ChIP-seq analysis of HilD, HilC, and RtsA, for the region encompassing *invF* and *invH*. Red arrows represent genes. RNA-seq graphs show sequence read density for one replicate experiment for cells lacking the indicated TF or for cells transiently overexpressing the indicated TF. ChIP-seq graphs show sequence read density for one replicate experiment for the indicated TF. The genes are colored red to indicate positive regulation by the TFs. Black arrowheads indicate ChIP-seq peaks. RNA-seq data are normalized according to the genome position with the 90th percentile for sequence read coverage.

HilD is considered the master regulator of *S*. Typhimurium invasion (8, 11). In a previous study, we mapped the binding of HilD across the *S*. Typhimurium genome using ChIP-seq (11). Thus, we identified 11 novel HilD-bound genomic regions. We showed that genes in four of these regions are associated with transcription activation by HilD. However, we assayed transcription activation by HilD in *Escherichia coli*, by fusing regions upstream of candidate genes to the *lacZ* reporter gene; failure to observe transcription activation by HilD in such experiments does not necessarily indicate that HilD does not regulate these genes, since factors specific to *Salmonella* may be required for their activation. To reassess the HilD regulon, we combined ChIP-seq and RNA-seq to map all direct regulatory targets of HilD in *S*. Typhimurium strain 14028s. For ChIP-seq, HilD was C-terminally epitope tagged and expressed from its native locus. For RNA-seq, we compared RNA levels in cells lacking *hilD* to those in cells transiently overexpressing HilD from a plasmid. We defined directly regulated genes as those protein-coding genes for which we observed a significant difference in RNA levels with and without HilD (see Table S1 in the supplemental material) and for which we detected binding by ChIP-seq (see Table S2) within a window extending from 600 bp upstream to 100 bp downstream of the annotated gene/operon start (see Table S3 and Materials and Methods for complete details on identification of directly regulated genes). An example of a directly HilD-regulated transcript is shown in Fig. 1.

**FIG 2:**
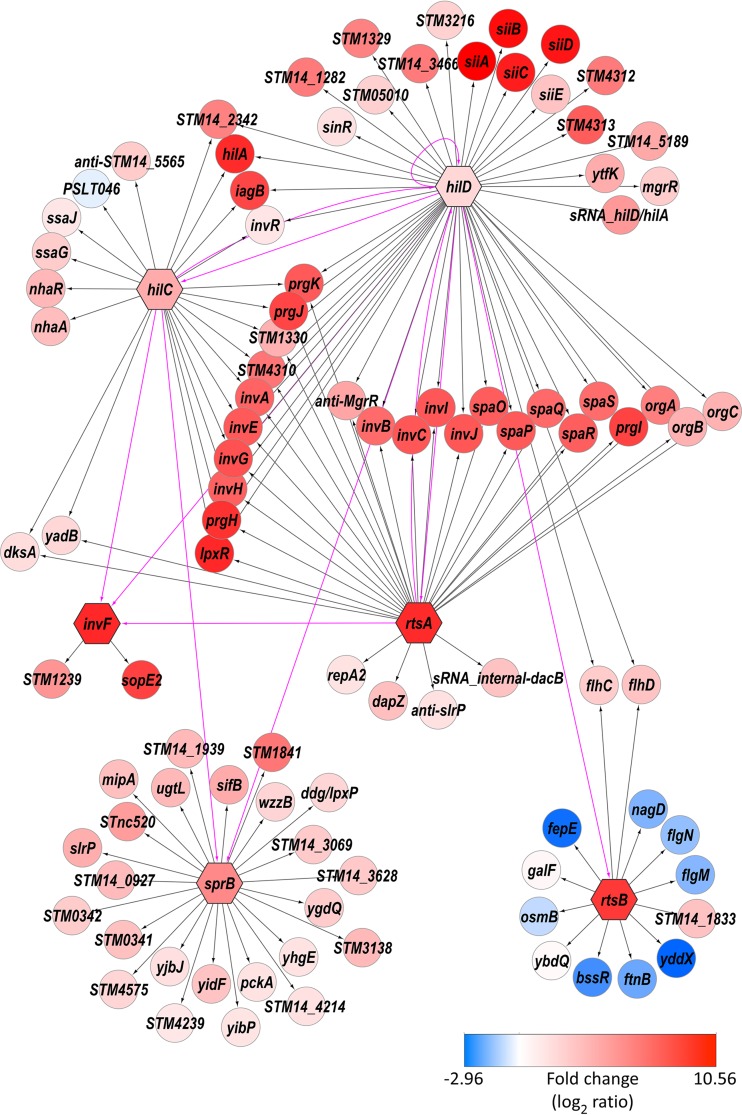
Regulatory network associated with SPI-1. The hexagon-shaped nodes represent TF-encoding genes *hilD*, *hilC*, *rtsA*, *invF*, *sprB*, and *rtsB*. The circular nodes represent target genes directly regulated by these TFs. The directed edges indicated with pink lines represent the regulatory relationships among the TFs. The directed edges indicated with black lines represent the regulatory relationships between the TFs and their target genes. The node color indicates the log ratio of the differential levels of expression (red = TF activated; blue = TF repressed), and the color scale is shown. Note that the regulatory targets of HilA are omitted because ChIP-seq for this protein was unsuccessful. Where possible, common gene names are shown. For genes without a common name, the name of the orthologous gene from *S*. Typhimurium strain LT2 is shown (“STMxxxx” names). For annotated genes without a common name and without an orthologue in *S*. Typhimurium strain LT2, the *S*. Typhimurium 14028s gene name is shown (“STM14_xxxx” names). For unannotated genes, we list a brief description of the transcript location (“anti” = antisense to the indicated gene; “sRNA_internal” = initiation within the indicated gene, in the sense orientation relative to that gene; “sRNA_hilD/hilA” = initiation in the intergenic region between *hilD* and *hilA*, antisense to the *hilA* 5′ UTR).

Most of the HilD binding events that we detected (see Table S2 in the supplemental material) corresponded to those described in our previous study (11). Nonetheless, our previous study included a few confirmed binding sites that we did not detect in the current study. This is likely due to differences in sensitivity between the sequencing approaches used, consistent with the missed targets all being in regions of low enrichment. We included these previously identified binding sites in our analysis of direct regulatory targets. All previously described direct regulatory targets of HilD were confirmed by our new analysis (Fig. 2). We identified several additional direct regulatory targets: *sinR* (see Fig. S1A), *STM14_1283* (*STM05010*) (for genes without common names, we list the homologous gene in *S. enterica* serovar Typhimurium strain LT2, assuming that an annotated homologue exists), *STM14_1613* (*STM1329*), *STM14_1614* (*STM1330*), *mcpC*, *STM14_5184* (*STM4310*), *STM14_5185* (*STM4312*), *STM14_5186* (*STM4313*), and *STM14_5189* (Fig. 2). Our previous study identified binding sites for HilD near the start codons of all of these genes (11), but we tested regulation of most using assays of reporter gene fusions in *E. coli* and did not observe transcription activation by HilD. We conclude that these genes require additional, *Salmonella*-specific factors for transcription activation by HilD.

### HilD directly regulates all other SPI-1-associated TFs.

Aside from HilD, four of the SPI-1-associated TFs, HilC, InvF, SprB, and HilA, are encoded within SPI-1. RtsA is a close homologue of HilD and HilC but is encoded outside SPI-1, in an operon that includes another TF-encoding gene, *rtsB*. Both *rtsA* and *rtsB* are strongly induced under conditions associated with SPI-1 expression (4). Our analysis of the HilD regulon indicated that HilD directly regulates all seven SPI-1-associated TFs, including HilD itself (Fig. 2) (11). This is consistent with the idea that HilD is the apex of a regulatory cascade associated with host cell invasion and that HilD activity is the primary target of environmental signals associated with invasion (9). We detected direct regulation of *sprB* by HilD from a binding site immediately upstream of *sprB*. Visual inspection of the RNA-seq data suggested that *sprB* is also cotranscribed with *hilC*, which is itself a direct HilD target (see Fig. S1B in the supplemental material). Using reverse transcription-PCR (rtPCR), we detected a transcript that spans the *hilC* and *sprB* genes (see Fig. S2). Given that the level of HilD binding upstream of *hilC* is substantially higher than that upstream of *sprB* (see Fig. S1B), we conclude that HilD regulation of *sprB* occurs predominantly through regulation of the *hilC*-*sprB* transcript.

### Combining ChIP-seq and RNA-seq to map the entire invasion regulon.

Although a few regulatory targets have been described for SPI-1-associated TFs other than HilD, only the HilD and HilA regulons have been determined on a genomic scale (11, 13). As described above, the ChIP-seq and RNA-seq approaches provide a complementary pipeline for identification of direct regulatory targets. Hence, we used this pipeline to identify the direct regulatory targets of HilC, RtsA, RtsB, InvF, and SprB (see Tables S1 to S3 in the supplemental material). For HilA, we were unable to detect robust ChIP-seq enrichment, suggesting that the epitope tags inactivated HilA function. We therefore limited our analysis of the HilA regulon to RNA-seq (see Table S1).

### The HilD, HilC, and RtsA regulons overlap but are distinct.

**FIG 3:**
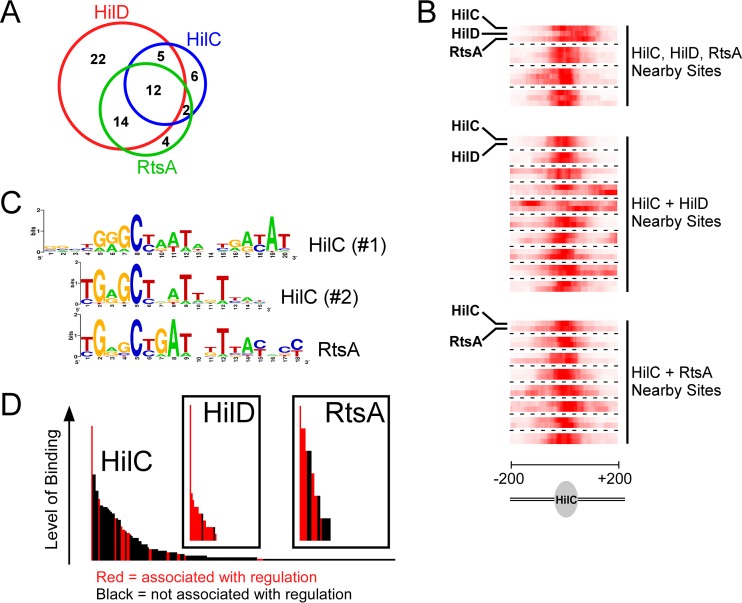
Overlap of the HilD, HilC, and RtsA regulons. (A) Venn diagram showing the degree of overlap of direct regulatory targets between HilD, HilC, and RtsA. (B) Heat maps showing overlap of ChIP-seq peaks for HilD, HilC, and RtsA. Each block shows data for two or three TFs at a single 400-bp genomic region, centered on a ChIP-seq peak for HilC. The combined, normalized sequence read count from the ChIP-seq data for HilC is shown in the first row of each block, with color intensity representing read density. The one or two additional rows in each block show equivalent data for HilD or RtsA or both, as indicated. (C) Sequence motifs for HilC and RtsA. (D) Association of ChIP-seq peaks with regulation for HilD, HilC, and RtsA. Each of the histograms represents all ChIP-seq peaks (i.e., binding sites for the corresponding TF). Each bar represents a single ChIP-seq peak, and the height of the bar indicates the level of binding (FAT score). Binding sites associated with regulation are indicated by red bars. Binding sites not associated with regulation are indicated by black bars.

HilC and RtsA are close homologues of HilD (7, 10, 11, 23). Previous work has shown that these TFs share many regulatory targets (10, 11, 21, 44). Using ChIP-seq and RNA-seq, we identified 21 direct target genes for HilC (12 transcripts) and 28 for RtsA (9 transcripts) (Fig. 2; see also Table S3 in the supplemental material). As expected, many of the HilD, HilC, and RtsA targets are shared (Fig. 1 and 3A). To determine whether HilD, HilC, and RtsA bind the same DNA sites upstream of shared target genes, we compared the profile of ChIP-seq reads across regions with closely positioned HilD/HilC/RtsA ChIP-seq peaks. The results of this analysis clearly indicate that HilD, HilC, and RtsA bind the same DNA sequences (Fig. 3B). Consistent with this, we were able to infer DNA sequence motifs for HilC (two similar motifs that are almost completely mutually exclusive in their site composition) and RtsA from the ChIP-seq data (Fig. 3C). Although the motifs are similar to each other, subtle differences in these motifs likely explain the overlapping but distinct binding profiles of HilC and RtsA. Although we could not derive a DNA sequence motif for HilD, it is likely that it binds with similar levels of sequence specificity to HilC and RtsA, given the overlap in target sites (Fig. 3B). Our data are consistent with an earlier study showing that HilC and HilD bind overlapping sites upstream of *hilA*, *hilD*, and *hilC* but that HilC binding is less sensitive to mutations within the DNA site (21).

Although the HilC regulatory targets were largely a subset of the HilD targets, HilC binds many more sites across the genome (209 sites for HilC versus 18 sites for HilD, not including the HilD sites from our previous study [11]). However, we detected regulation of nearby genes for only a small fraction of the HilC binding sites (Fig. 3D; see also Fig. S1C in the supplemental material). In contrast, we detected regulation of nearby genes for all but two HilD binding sites and for 50% of the RtsA binding sites (we include regulation of noncoding RNAs, discussed below; Fig. 3D). These data suggest that HilC has a more relaxed DNA sequence specificity than HilD or RtsA, but that HilC is a less potent activator of transcription.

Transcription activation of SPI-1 genes by HilD, HilC, and RtsA is believed to be due to countersilencing of repressive NAPs H-NS and Hha rather than to direct interaction between the activators and initiating RNA polymerase (29, 30). We compared the locations of HilD, HilC, and RtsA binding sites to the location of H-NS occupancy, as determined by chromatin immunoprecipitation with microarray technology (ChIP-chip) analysis performed using the closely related LT2 strain of *S*. Typhimurium (26). Five of the HilD/HilC/RtsA-bound regions associated with direct transcription activation of protein-coding genes outside SPI-1 overlap regions that are likely bound by H-NS (HilD/HilC/RtsA binding sites associated with regulation of *lpxR*, *STM14_5184*, *siiA*, *mcpC*, and *ssaG*). Thus, many of the target genes of HilD, HilC, and RtsA are likely regulated by a countersilencing mechanism. This is consistent with the fact that *hilD*, *hilC*, and *rtsA* were horizontally acquired by *S. enterica* (45) and that countersilencing is expected to evolve more quickly than other mechanisms of transcription activation (36).

### InvF and SprB broaden the extent of positive regulation associated with invasion.

HilD, HilC, and RtsA all positively regulate *invF*, which is located within SPI-1 and encodes another of the SPI-1-associated TFs (Fig. 1 and 2). Three regulatory targets of InvF, *sopB* (*sigD*), *sicA*, and *sopE*, have been described previously (14, 46) (note that *sopE* is not present in the strain we used but that a close homologue, *sopE2*, is present). *sopB* and *sopE2* encode SPI-1-secreted proteins, and *sicA* encodes a chaperone for SPI-1 secreted proteins. *sopB* and *sopE2* are located outside SPI-1. Our ChIP-seq and RNA-seq data for InvF confirm direct regulation of *sopE2* (Fig. 2; see also Fig. S1D in the supplemental material) and identify *STM14_1486* (*STM1239*; Fig. 2; see also Fig. S1E) as an additional gene that is positively regulated by InvF. Given that all other described InvF targets are directly involved in the function of the SPI-1-encoded T3SS, our data strongly implicate STM14_1486 in this process. Surprisingly, we did not detect InvF binding upstream of *sopB* or *sicA*, despite these genes having been previously shown to be regulated directly by InvF (14). Our RNA-seq data show strong upregulation of *sopB* and *sicA* by InvF (see Table S1), suggesting that failure to detect binding of InvF by ChIP-seq might be due to partial inactivation of the protein by the epitope tags. This seems unlikely given that we detected robust association of InvF with sites upstream of *sopE2* and *STM14_1486* (see Fig. S1D and E). An alternative explanation is that InvF binds upstream of *sopB* and *sicA* only when present at high concentrations in the cell, as would be the case in our RNA-seq experiment in which InvF was transiently overexpressed. Since we identified only two regions bound by InvF, we were not able to infer the sequence of its cognate DNA sites. However, a consensus sequence for InvF has been described previously (46), and a similar sequence is found upstream of *sopE2* and *STM14_1486*, coincident with the InvF-enriched genomic region (see Fig. S3).

HilD and HilC positively regulate *sprB*, most likely cotranscriptionally with *hilC* itself, since *hilC* and *sprB* are cotranscribed (see Fig. S2 in the supplemental material). The only previously described target of SprB is the *siiABCDEF* operon of SPI-4 (24). Our ChIP-seq and RNA-seq data identified 23 direct target genes for SprB, all of which are positively regulated. An example of an SprB-regulated gene, *STM14_2227* (*STM1841*), is shown in Fig. S1F. Strikingly, we detected no significant regulation of SPI-4 genes (see Table S1), and we detected no binding of SprB within or near SPI-4 (see Table S2 and Fig. S1G). We conclude that SPI-4 is not a regulatory target of SprB. We and others have shown that HilD and HilA positively regulate SPI-4 genes (10, 11, 15, 17), explaining how SPI-4 gene expression is coordinated with that of SPI-1.

### RtsB is a negative regulator with a regulon overlapping that of RcsB.

RtsA and RtsB are encoded by genes that are next to one another and are predicted to be cotranscribed in an operon (10). Both *rtsA* and *rtsB* are strongly induced under conditions associated with activation of SPI-1 genes (4). Consistent with this, the *rtsAB* operon is a direct regulatory target of HilD (Fig. 2). Our data also indicate that the two genes downstream of *rtsB* are likely to be cotranscribed with *rtsAB* and that the nearby *STM14_5184* gene is positively regulated by HilD, HilC, and RtsA (Fig. 2). Thus, although this genomic region falls well outside SPI-1, the genes it contains are coordinately regulated with those in SPI-1. RtsB is a TF that includes a DNA-binding domain belonging to the family of two-component response regulators. However, in contrast to most members of this family, RtsB lacks a receiver domain, suggesting that it cannot directly respond to signaling from a histidine kinase. Our ChIP-seq and RNA-seq data identified 10 direct regulatory targets of RtsB (Fig. 2). With one exception, all these genes are negatively regulated by RtsB. This is in contrast to most two-component system regulators, which predominantly function to activate transcription. We observed negative regulation of the *flhDC* operon that encodes the master regulators of flagellar motility (see Fig. S1H in the supplemental material), consistent with a previous study (10). We also observed negative regulation of *flgM* and *yddX* by RtsB (Fig. 2). *flgM* encodes an anti-sigma factor for FliA (σ^28^), the flagellar Sigma factor (47). *yddX* encodes a positive regulator of flagellar synthesis in *E. coli* (48). Thus, RtsB both negatively (through FlhDC and YddX) and positively (indirectly, through FlgM) regulates expression of flagellar motility genes. In contrast, HilD positively regulates FlhDC (see Fig. S1H), suggesting that flagellar regulation during invasion is complex.

Strikingly, the most enriched region in the RtsB ChIP-seq data, as well as five other major ChIP-seq peaks, was upstream of the *std* operon that encodes a fimbrial apparatus whose expression is connected to that of SPI-1 genes (see Fig. S1I in the supplemental material) (49). However, we detected no significant difference in the levels of expression of *std* genes between cells lacking *rtsB* and cells transiently overexpressing RtsB (see Fig. S1I and Table S1). Given the number and strength of the binding sites for RtsB upstream of the *std* operon, we propose that RtsB regulates transcription of these genes under a growth condition different from that used in our study.

**FIG 4:**
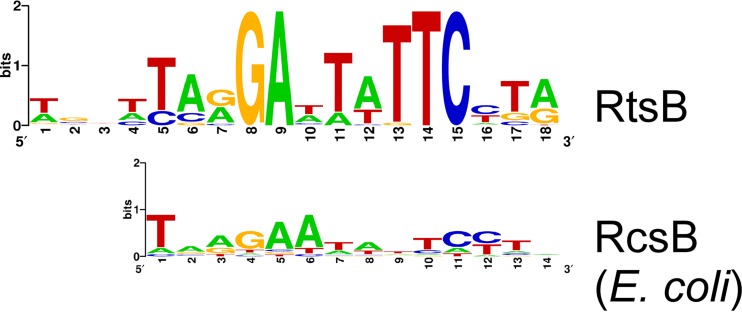
RtsB binding sites that overlap those of RcsB. Data represent results of comparison of the sequence motif for RtsB to the sequence motif for *E. coli* RcsB taken from the PRODORIC database (90).

As for HilC and RtsA, we were able to infer a DNA motif for RtsB binding by searching for enriched sequences within the RtsB-bound regions (Fig. 4). This motif closely resembles that of *E. coli* RcsB, a two-component system regulator (Fig. 4). RcsB is known to repress transcription of *flhDC* in both *E. coli* (50) and *S. enterica* (19). Moreover, RcsB regulates *yddX* in *E. coli* (51). Thus, our data suggest regulatory interplay between RcsB and RtsB. Intriguingly, RcsB has been shown previously to regulate genes in the *std* operon (52), further suggesting that RtsB regulates this operon.

### Redefining the HilA regulon using RNA-seq data.

HilA is known to positively regulate the transcripts of *inv* and *spa*, *sic* and *sip*, and *prg* and *org* located within SPI-1 (8), and a combined ChIP-chip and microarray transcription profiling study identified additional HilA targets (13). We failed to detect any binding sites for HilA by ChIP-seq, suggesting that the epitope tags interfered with HilA function. However, RNA-seq data from a Δ*hilA* strain with/without transient overexpression of HilA were consistent with regulation of transcripts within SPI-1 (see Table S1 in the supplemental material). Strong regulation of genes outside SPI-1 was observed, but most of these genes are direct regulatory targets of other SPI-1-associated TFs (HilD, HilC, RtsA, InvF, and SprB), and HilA likely regulates expression of one or more of these TFs. Nonetheless, we were able to identify likely direct targets of HilA based on the criteria that (i) the genes were strongly regulated in the RNA-seq experiments and (ii) the genes are not direct regulatory targets of other known SPI-1-associated TFs. Thus, we identified genes in the *pspA*-*pspD* operon and *pspG* as likely HilA targets (see Fig. S1J). These genes encode phage shock proteins that are essential for virulence, likely because of their role in maintaining proton motive force during intracellular growth (53). We also identified *yiaD* (see Fig. S1K) and *STM14_1174-8* (*STM1034*-*8*) as likely direct targets of HilA. Surprisingly, the 13 genes located outside SPI-1 that have been previously reported as direct HilA targets (13) were not significantly regulated in our RNA-seq experiment. One of the previously reported HilA targets for which we did not observe significant regulation is *flhD* (13). We note that two previous studies provided strong evidence that *flhD* is not regulated by HilA (18, 54). We therefore suggest that the previously reported HilA targets outside SPI-1 (13) likely represent false positives.

### Regulation of noncoding RNAs by SPI-1-associated TFs.

Bacterial genomes encode large numbers of small RNAs (sRNAs) that are typically noncoding (55). sRNAs often function as regulators of mRNA stability and/or translation by base pairing with specific mRNA targets (55). Many sRNAs have been identified in *S*. Typhimurium (56–59). Two sRNAs, InvR and DapZ, have been described previously as direct regulatory targets of HilD (56, 60). We took an unbiased approach to determine whether these and any additional sRNAs are regulated by SPI-1-associated TFs. We applied the Rockhopper RNA-seq analysis tool to our RNA-seq data for each TF, in the absence of any reference genome sequence (61). This method simultaneously assembles transcript fragments (“transfrags”; see Materials and Methods) (62) from the raw RNA-seq data and identifies those transfrags that are significantly differentially expressed between cells lacking a TF and cells transiently overexpressing a TF. Note that a single mRNA can be represented by multiple nonoverlapping transfrags since it is often not possible to assemble a complete transcript sequence from RNA-seq data alone. Regulated transfrags were mapped back to the 14028s genome. We then selected any transfrag whose 5′ end fell <200 bp downstream or <100 bp upstream of a TF binding site identified by ChIP-seq. The majority of these directly regulated transfrags corresponded to the 5′ portion of mRNAs for protein-coding genes identified in our conventional analysis pipeline. However, we identified several additional directly regulated transfrags. These correspond to known sRNAs, novel sRNAs, and 5′ untranscribed regions (UTRs) of genes that were not detectably regulated in our conventional analysis. A summary of sRNAs directly regulated by SPI-1-associated TFs is shown in Table 1.

**TABLE 1:** List of regulated noncoding RNAs identified in this study

Start (bp)^a,b^	Stop (bp)^a,b^	Strand	Description	TF regulator(s)	Fold change^c^
34013*	33811*	−	Antisense to *STM14_5565*	HilC	2.28^d^
74905	74971	+	DapZ	RtsA	2.66
868925	868081	−	Antisense to *slrP*	RtsA	1.25
1343788	1343659	−	STnc520	SprB	4.08
1418433	1418523	+	Overlapping *STM14*_*1613*/*STM14*_*1614*	HilC	1.18
1603873	1604092	+	MgrR	HilD	2.12
1603938	1603781	−	Overlapping *STM14_1833*	HilD, RtsA	3.73, 2.05^e^
3039507	3039347	−	Antisense to *hilA* 5′ UTR	HilD	4.23
3065151	3066061	+	InvR	HilD, HilC	1.07, 3.99^e^
3483808	3483985	+	Within *dacB*	RtsA	2.54

a The numbers represent predicted start/stop genome coordinates for a given noncoding RNA (ncRNA). Numbers marked with an asterisk indicate RNAs encoded on the virulence plasmid.

b In some cases, multiple, overlapping fragments of an ncRNA were identified, or overlapping ncRNAs were identified for multiple TFs. These instances were merged into a single unit.

c Data represent fold change (log_2_) in RNA level for cells showing overexpression of the corresponding TF versus cells in which the TF-encoding gene was deleted.

d Data represent averages of fold change values for three overlapping transfrags.

e The two values correspond to the 2 TFs, in the order listed in the previous column.

Consistent with a previous study (60), we observed that the InvR sRNA is a direct regulatory target of HilD (Fig. 2). We also observed direct, positive regulation of InvR by HilC (Fig. 2). We did not observe direct regulation of the DapZ sRNA by HilD, in contrast to a previous report (56). Rather, we observed direct regulation of DapZ by RtsA (see Fig. S1L in the supplemental material). Moreover, while we did not detect a HilC-regulated transfrag for DapZ using Rockhopper, we did detect strong binding of HilC upstream of *dapZ* (see Fig. S1L), and visual inspection of the RNA-seq data (see Fig. S1L) strongly supports the idea of direct regulation by HilC. We propose that DapZ is regulated by both RtsA and HilC but not HilD. This model is consistent with a previous study which showed a larger decrease in DapZ levels in cells lacking HilD than in cells lacking either HilC or RtsA (56). Since HilD positively regulates both HilC and RtsA (Fig. 2) (11, 20, 21), loss of HilD would be expected to result in a larger decrease in DapZ expression than loss of either HilC or RtsA individually.

**FIG 5:**
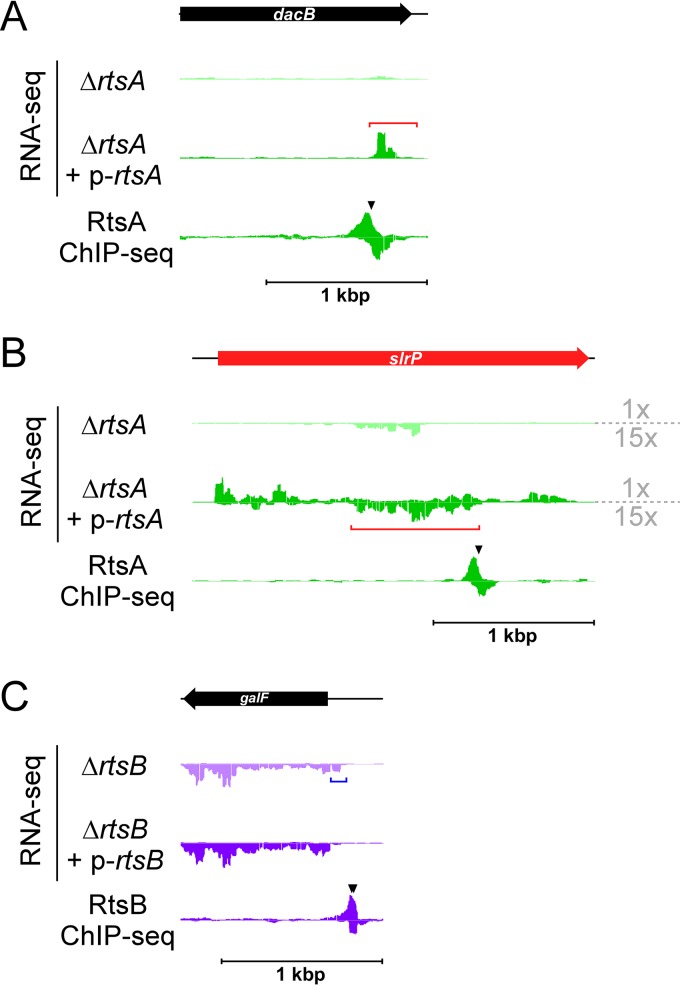
Identification of regulated sRNAs and 5′ UTRs. (A) Data represent results of RNA-seq and ChIP-seq analysis of RtsA, for the region encompassing *dacB*. The sRNA that initiates within *dacB* is indicated by a red bracket. (B) RNA-seq and ChIP-seq data for RtsA, for the region encompassing *slrP*. The sRNA that initiates antisense to *slrP* is indicated by a red bracket. Note that the RNA-seq data for the minus strand are zoomed 15 times relative to the data for the plus strand. (C) RNA-seq and ChIP-seq data for RtsB, for the region encompassing *galF*. The 5′ UTR of a *galF* transcript that is regulated by RtsB is indicated by a blue bracket. In all panels, the genes are colored red or black to indicate positive regulation or no regulation by the TFs. Black arrowheads indicate ChIP-seq peaks.

Few of the sRNAs regulated by SPI-1 TFs (Table 1) have been described previously (59). Four of the regulated sRNAs are transcribed from loci that do not overlap other annotated genes. These include an sRNA that overlaps considerably with the previously described STnc520, whose expression was shown to be strongly induced under conditions associated with activation of SPI-1 (4). This is consistent with our observation that the STnc520-overlapping sRNA is positively regulated by SprB (Fig. 2 and Table 1). We detected regulation of two sRNAs that overlap the 3′ ends of protein-coding genes in the sense orientation. One of these is DapZ. The other, an RtsA target, is a novel sRNA that initiates within *dacB* (Fig. 5A). These data are consistent with the recent observation of sense-orientation sequence within protein-coding genes being a rich source of sRNAs (56). We also identified three antisense sRNAs. One of these, a target of HilD and RtsA, is antisense to an sRNA, MgrR, which itself is directly regulated by HilD. A particularly intriguing antisense RNA initiates within *slrP* and is positively regulated by RtsA from a binding site within *slrP* (Fig. 5B). This RNA has been observed previously (4), but regulation has not been described. Strikingly, increased expression of the antisense RNA within *slrP* correlates with increased expression of *slrP* itself; *slrP* mRNA levels are 39-fold higher in cells transiently overexpressing RtsA than in cells lacking *rtsA* (Fig. 5B). Regulation of *slrP* by RtsA has been described previously (10), but the reporter gene fusion used in that study lacked the region within *slrP* that is bound by RtsA. We propose that the previously observed regulation of *slrP* by RtsA (10) occurs indirectly, via SprB (Fig. 2), and that an additional level of regulation by RtsA occurs via the antisense RNA within the *slrP* gene.

### Regulation of transcript variants by SPI-1-associated TFs.

We detected regulated transfrags for several regions that are immediately upstream of protein-coding genes, on the same strand. In some cases, these were disregarded because the upstream gene was identified in our standard RNA-seq analysis as being a direct regulatory target of the same TF. One example of the latter, upstream of *invF*, was notable because the RNA initiates well within the *invH* gene, in the antisense orientation. This transfrag is regulated by HilD and HilC. Our RNA-seq data suggested that this RNA is the 5′ UTR for the transcript that begins with *invF* (Fig. 1), consistent with earlier studies (12, 63). We confirmed this using rtPCR (see Fig. S4 in the supplemental material). Thus, expression of the *invF* transcript may also be associated with regulation of *invH* due to the high potential for base pairing between these RNAs. Four other regulated transfrags, three regulated by RtsB and one by HilC, are immediately upstream of genes not significantly regulated according to our standard RNA-seq analysis (see Table S4). An example of such a gene, *galF*, is shown in Fig. 5C. In these cases, we suspected that the genes have multiple transcription start sites, with the most upstream promoter being regulated by the TF in question. Thus, the regulated transcript is a variant with a longer 5′ UTR. Published transcription start site mapping supports the idea of multiple transcription start sites for two of the genes, *osmB* and *galF* (4). We confirmed the presence of a long 5′ UTR for all four genes by the use of rtPCR (see Fig. S4). Although the overall RNA levels for these genes were not significantly affected by RtsB expression under the conditions that we used (see Table S1), we propose that significant regulation might be observable under other growth conditions, or that the regulated transcript variants might have altered translation efficiency due to their extended 5′ UTRs.

### An expanded regulatory network for invasion.

**FIG 6:**
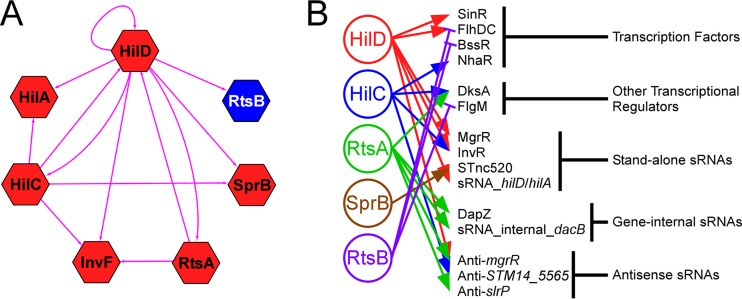
Extensive cross talk between SPI-1 regulators, and predicted network expansion. (A) The hexagon-shaped nodes represent TF genes *hilD*, *hilC*, *rtsA*, *hilA*, *invF*, *sprB*, and *rtsB*. The directed edges indicated with pink lines represent the regulatory relationships among these TFs. The node color indicates whether the TF is a positive (red) or negative (blue) regulator. (B) Regulation of known and predicted regulators by SPI-1 TFs. Positive regulation is indicated by lines with arrowheads. Negative regulation is indicated by lines with flat heads.

Cross talk between regulatory pathways is a common theme in bacterial gene regulation (64). Our data demonstrate extensive cross talk in the SPI-1 regulatory network (Fig. 6A). In particular, HilD directly regulates expression of all 7 TFs, including itself. Moreover, as has been described previously, the HilD, HilC, and RtsA regulons overlap extensively (11, 22). In addition to the known SPI-1-associated TFs, our data suggest the involvement of many other regulatory molecules in the gene expression changes that accompany invasion (Fig. 6B). We observed direct regulation of 4 TFs (FlhDC, BssR, SinR, and NhaR) as well as of two other proteins that regulate transcription (FlgM and DksA). We also observed direct regulation of many sRNAs. Some of these sRNAs (e.g., MgrR) are known regulators, whereas others have not been studied previously. Nonetheless, most sRNAs studied to date have been shown to regulate expression of other genes through base-pairing interactions (55). Hence, we propose that *Salmonella* invasion is associated with widespread regulation by sRNAs.

### A core set of invasion genes.

**FIG 7:**
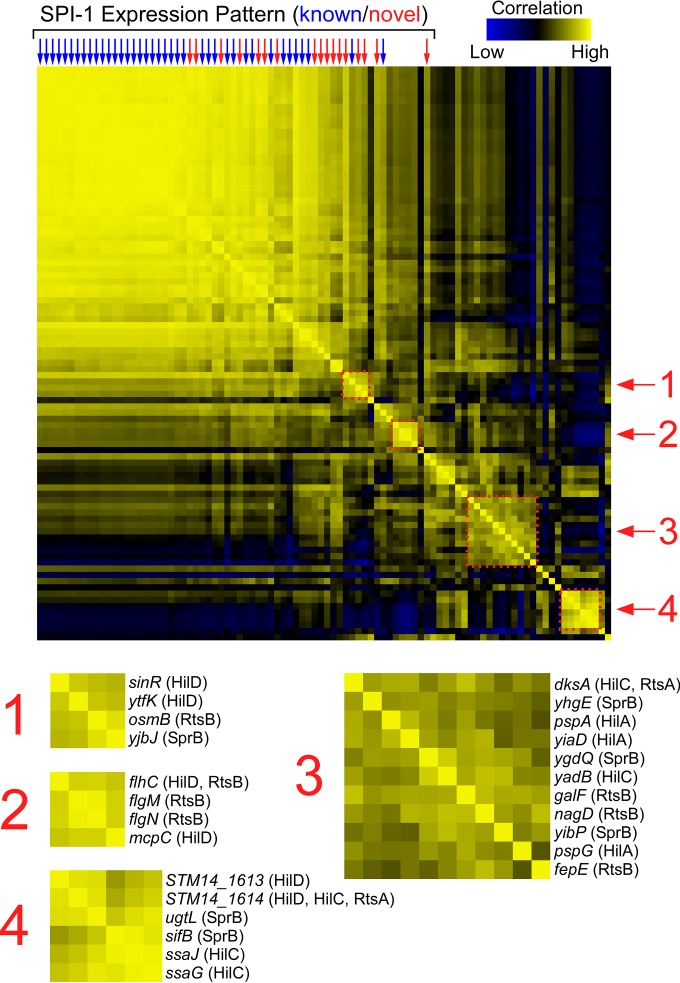
Hierarchical clustering of gene expression profiles reveals connections between genes regulated by SPI-1-associated TFs. The central heat map shows pairwise correlations between gene expression profiles (data from reference 4) for every direct regulatory target of HilD, HilC, RtsA, InvF, SprB, RtsB, and HilA. Each row/column represents a gene, with genes arrayed in identical orders in rows and columns (note the symmetry of the heat map). Stronger yellow colors indicate higher correlation coefficients, as indicated on the scale (bottom right). Columns indicated by arrows represent genes whose expression profile has an average correlation coefficient of >0.5 for comparisons to all genes located within SPI-1. Blue arrows indicate known invasion genes. Red arrows indicate likely novel invasion genes. The four dashed squares highlight clusters of genes whose expression profiles correlate strongly. These clusters are enlarged and annotated below the heat map.

A recent study used RNA-seq to determine expression changes across 22 different growth conditions (4). Using this resource, we selected the expression profiles, for the 22 tested growth conditions, for each of the SPI-1-associated TF target genes. We then grouped the expression profiles using hierarchical clustering. Thus, we were able to identify pairs of genes with similar expression profiles across the 22 growth conditions. Lastly, we compared the expression profiles for each gene by determining correlation coefficients of the 22 expression values for each gene pair. Figure 7 shows the complete set of pairwise correlation coefficients. Each row or column represents a gene identified in our study, and the shading at each row/column intersect indicates how well the expression profiles for the corresponding two genes correlate. Thus, yellow blocks indicate pairs of genes with similar expression levels across the 22 growth conditions analyzed. As expected, known invasion genes formed a tightly clustered group with highly correlated gene expression profiles (Fig. 7). Strikingly, many of the novel regulatory targets identified in our study displayed highly correlated gene expression profiles with the known invasion genes. Specifically, we identified 17 novel regulatory targets encoded outside SPI-1 whose expression profiles have an average correlation coefficient of >0.5 with the profiles for invasion genes within SPI-1 (Fig. 7 and Table 2). These genes include regulatory targets of multiple TFs, notably, SprB, InvF, and HilA. Given that these 17 genes are direct regulatory targets of SPI-1-associated TFs and that their expression profiles are very similar to those of known invasion genes, we conclude that most or all of these genes are directly involved in the invasion process. Addition of these genes would greatly expand the set of known invasion genes.

**TABLE 2:** List of putative novel invasion genes

Gene ID^a^	Common name^a^	ST4/74 name^b^	LT2 name^c^	TF^d^
*STM14_0358*	*sinR*	*SL0300*	*STM0304*	HilD
*STM14_5184*		*SL4247*	*STM4310*	HilD/HilC/RtsA
*STM14_5185*		*SL4248*	*STM4312*	HilD
*STM14_5186*		*SL4249*	*STM4313*	HilD
*STM14_0048*	*nhaA*	*SL0040*	*STM0039*	HilC
*STM14_0049*	*nhaR*	*SL0041*	*STM0040*	HilC
*STM14_1486*		*SL1177*	*STM1239*	InvF
*STM14_0398*		*SL0336*	*STM0341*	SprB
*STM14_2227*		*SL1770*	*STM1841*	SprB
*STM14_3799*		*SL3112*	*STM3138*	SprB
*STM14_4215*	*pckA*	*SL3467*	*STM3500*	SprB
*STM14_5097*	*yjbJ*	*SL4176*	*STM4240*	SprB
NA	*STnc520*	*STnc520*	*STnc520*	SprB
*STM14_1174*		*SL0973*	*STM1034*	HilA
*STM14_1176*		*SL0975*	*STM1036*	HilA
*STM14_1177*		*SL0976*	*STM1037*	HilA

a The genes listed are direct regulatory targets of SPI-1-associated TFs that are encoded outside SPI-1 whose expression profiles (from reference 4) have an average correlation coefficient of >0.5 with the profiles for invasion genes within SPI-1.

b Gene name for homologue in strain ST4/74.

c Gene name for homologue in strain LT2.

d The listed TF(s) directly regulates the corresponding gene.

### Connecting novel invasion genes with virulence.

Given the importance of the invasion process for infection of host cells, it is perhaps unsurprising that many genes within SPI-1 have been shown to be required for establishing a productive infection in a variety of different model systems, both *in vitro* (i.e., cultured mammalian cells) and *in vivo* (mouse models of infection) (65–68). We analyzed published datasets to determine whether any of the novel invasion genes were identified as being important for productive infection of pigs, chicks, or cows by the use of an oral model of infection (68). Note that other infection models, such as intraperitoneal infection, are less likely to be dependent upon invasion. Of the 17 novel invasion genes, mutants of 14 were analyzed for their ability to infect each of the three hosts. Strikingly, the number of instances where mutants with mutations in these 14 genes were found to be defective in infection was far greater than that expected by chance (binomial test *P* = 4.7E^−9^). Specifically, mutants with mutations in nine of the novel invasion genes tested (*STM14_5186*, *STM14_5184*, *STM14_5185*, *STM14_3799* [*STM3138*], *STnc520*, *STM14_1486*, *nhaA*, *sinR*, and *mcpC*) were defective in establishing an infection in all three infection models (68), and mutants with mutations in *STM14_2227* were defective in infection of chicks and cows but were not tested in pigs (68). These data suggest that most of the 17 novel invasion genes are directly or indirectly involved in invasion and that their regulation by SPI-1-associated TFs plays an important role in coordinating invasion processes.

### Regulatory cross talk groups functionally related SPI-1-associated TF target genes.

Hierarchical clustering of gene expression profiles for the SPI-1-associated TF target genes revealed multiple smaller groups of genes with highly correlated expression profiles. Four such clusters are highlighted in Fig. 7. For one of the clusters (cluster 2), the four genes, *flhC*, *flgM*, *flgN*, and *mcpC*, are direct targets of either HilD or RtsB (*flhC* is regulated by both; Fig. 7). Despite being regulated by two different TFs, these genes exhibit similar expression profiles, strongly suggesting an additional, shared regulatory input. For this specific example, the shared regulatory input is known: these genes are all involved in motility and chemotaxis, and their expression is known to be controlled directly or indirectly by the master regulator of flagellar synthesis, FlhDC (69). Given that a connection between SPI-1 and flagellar regulation is well established (19), this cluster validates our approach, indicating that other clusters are very likely to have shared regulatory input that is independent of SPI-1. Cluster 4 (Fig. 7) is particularly interesting because three of the genes in this cluster are associated with secretion by the Salmonella pathogenicity island 2 (SPI-2) T3SS that secretes proteins required for intracellular survival and persistence (70): *ssaG* and *ssaJ* encode core components of the SPI-2 T3SS machinery (71, 72), and *sifB* encodes a secreted effector protein (73). *ssaG* and *ssaJ* are regulated by HilC, and *sifB* is regulated by SprB. Thus, our data indicate cross talk between SPI-1 and SPI-2 regulation mediated by HilC and SprB. Moreover, our data indicate a connection between SPI-1 and SPI-2 through three additional genes: *ugtL*, *STM14_1613*, and *STM14_1614*. Strikingly, *ugtL*, a target of SprB, is also regulated by SsrB, the master regulator of SPI-1 (74), reinforcing the idea of a role of this gene in cross talk between the two T3SSs. Although expression of SPI-1 and SPI-2 genes is believed to occur at different stages of the infection process, cross talk between the two has been previously described. Specifically, HilD is believed to activate transcription of the genes encoding the SpiR(SsrA)/SsrB two-component regulatory system that is the master regulator of SPI-2 (75). Our data indicate that cross talk between SPI-1 and SPI-2 is more extensive than previously appreciated.

As described above, our hierarchical clustering analysis revealed groups of genes with shared regulatory inputs, which in turn likely indicates shared functions. While it is not immediately clear what the shared regulatory input and shared functions are (e.g., clusters 1 and 3 in Fig. 7), our data will guide future studies of these gene clusters. Moreover, function can be inferred in some cases from the activity of other gene members within a cluster, such as cross talk between SPI-1 and SPI-2 for genes in cluster 4 (Fig. 7). Strikingly, each of the gene clusters includes regulatory targets of different SPI-1-associated TFs. This indicates that cross talk with other regulatory pathways is broadly associated with invasion TF regulation rather than with specific, individual TFs.

### A community resource for *Salmonella* gene regulation.

Our data represent the first global analysis of regulation associated with *Salmonella* invasion. The regulatory network that we describe is highly interconnected, especially in the HilD/HilC/RtsA module, and likely extends well beyond its currently defined gene set due to the regulation of many known and putative regulators by SPI-1 TFs. Analysis of gene expression profiles for genes within the network has revealed a large set of genes that are likely to be novel invasion genes, and facilitated identification of functionally related genes based on their shared expression profiles. We anticipate that our data will provide a valuable resource to other groups studying *Salmonella* virulence. Hence, we have made our data freely accessible at http://www.wadsworth.org/research/scientific-resources/interactive-genomics or http://salmonella.wadsworth.org using JBrowse, a web-based browser for visualizing genome-scale datasets (76).

## MATERIALS AND METHODS

### Strains and plasmids.

All strains and plasmids used in this work are listed in Table S5 in the supplemental material. All oligonucleotides used in this work are listed in Table S6. DNA sequence manipulation was performed using BioWord (77). Genomic analyses for 14028s used NCBI reference sequence NC_016856.1.

All strains of *Salmonella enterica* subspecies *enterica* serovar Typhimurium used in this work were 14028s strains (78). Three C-terminal FLAG tags were inserted chromosomally for *hilD*, *hilC*, *rtsA*, *invF*, *sprB*, *rtsB*, and *hilA*, using the FRUIT method, as previously described (79), to yield strains AMD475, AMD474, AMD476, AMD478, AMD508, AMD477, and AMD473, respectively. Deletions of *hilD*, *hilC*, *rtsA*, *invF*, *sprB*, *rtsB*, and *hilA* were constructed by P22 transduction from existing deletion strains to yield strains CDS024, CDS022, CDS020, CDS028, CDS026, CDS030, and CDS032, respectively. Both the donor and recipient strains were 14028s or derivatives of 14028s. Note that we chose to recreate these mutations by P22 transduction to eliminate the possibility that the strains contained any other differences from one another or from the parent wild-type strain.

Plasmids pBLP013, pBLP011, and pBLP010 have been described previously (11) and are derivatives of pBAD24 (80) that express *hilD*, *hilC*, and *rtsA*, respectively, from the P_BAD_ promoter. pCDS001, pCDS002, pCDS003, and pCDS004 are derivatives of pBAD24 (80) that express *rtsB*, *sprB*, *invF*, and *hilA*, respectively, from the P_BAD_ promoter. These plasmids were constructed by amplifying the corresponding genes from strain 14028s by colony PCR using oligonucleotides listed in Table S6 in the supplemental material, followed by insertion into pBAD24 cut with NcoI using an In-Fusion kit (Clontech).

### ChIP-seq.

Strains AMD475, AMD474, AMD476, AMD478, AMD508, and AMD477 (14028s with C-terminally FLAG-tagged *hilD*, *hilC*, *rtsA*, *invF*, *sprB*, and *rtsB*, respectively) were used for ChIP-seq. Cells were grown overnight in LB (0.17 M NaCl), subcultured 1 in 50 in high-salt LB (0.3 M NaCl), and shaken at 50 rpm at 37°C for 5.5 h. ChIP-seq was performed using M2 anti-FLAG antibody (Sigma), as described previously (81). Two biological replicates were performed for each ChIP-seq experiment.

### ChIP-seq peak identification.

Identification of peaks from the ChIP-seq data was performed as described previously (81), except that the shift between forward and reverse strands was assumed to be at least 20 nucleotides (20 nt). All ChIP-seq peaks are listed in Table S2 in the supplemental material.

### Motif identification and scoring.

To identify enriched sequence motifs from ChIP-seq data, we first extracted 100-bp sequences centered on each of the ChIP-seq peaks. In cases where these sequences overlapped, we merged them into a single sequence of >100 bp. We then used MEME-ChIP (with the default parameters, except that the “any number of sequences” option was selected) to identify enriched sequence motifs. Only motifs with an *E* value of <0.01 are reported.

### RNA-seq.

Strains CDS024, CDS022, CDS020, CDS028, CDS026, CDS030, and CDS032 (14028s strains with an unmarked deletion of *hilD*, *hilC*, *rtsA*, *invF*, *sprB*, *rtsB*, and *hilA*, respectively) were used for RNA-seq. These strains were transformed either with empty pBAD24 or with pBAD24 derivatives pBLP013, pBLP011, pBLP010, pCDS003, pCDS002, pCDS001, and pCDS004, which express *hilD*, *hilC*, *rtsA*, *invF*, *sprB*, *rtsB*, and *hilA*, respectively (the pBAD24-encoded TF matched the deleted TF gene for each strain). Cells were grown overnight in LB (0.17 M NaCl), subcultured 1 in 50 in high-salt LB (0.3 M NaCl), and shaken at 50 rpm at 37°C for 5.5 h, and arabinose was added (0.2% final concentration) for 7 min before harvesting was performed. Note that 7 min was chosen as the induction time because a previous study had showed that expression of a regulator leads to direct regulatory effects that occur between 5 and 10 min after induction (82) and because transient induction of TF expression limits indirect regulatory effects. RNA purification, DNase I treatment, rRNA removal, and Illumina library construction were performed as described previously (83). Two biological replicates were performed for each RNA-seq experiment.

### Identification of direct regulatory targets of SPI-1-associated TFs using genome sequence and annotation.

The following procedure was applied for each of HilD, HilC, RtsA, InvF, SprB, and RtsB. RNA-seq data were analyzed using Rockhopper (version 2.02, default parameters) (84), comparing expression of TF-deleted cells with results determined using empty vector and cells transiently overexpressing the TF. Based on this analysis, annotated genes were selected if their RNA levels were significantly different (*q* = <0.01) by >2-fold in cells lacking the TF and cells in which the TF was transiently expressed. All RNA-seq analysis data generated using a reference genome are listed in Table S1 in the supplemental material. We then compared the positions of the starts of these regulated genes to the positions of ChIP-seq peaks for the corresponding TF. Significantly regulated genes were classified as “direct regulatory targets” if the start codon of the gene was determined to be ≤600 bp downstream or ≤100 bp upstream of a ChIP-seq peak. All direct regulatory targets, and associated metadata, are listed in Table S3.

### Network analysis.

The gene regulatory network graph was constructed and visualized using Cytoscape software (version 3.1.1) (85). TF genes *hilD*, *hilC*, *rtsA*, *invF*, *sprB*, and *rtsB* were assigned as source nodes, and their target genes were assigned as target nodes. The regulatory relationships between the TFs and their target genes were represented by directed edges. Node identity is double-encoded as shape, indicating source or target nodes, and color, indicating the level of regulation. For target genes regulated by multiple TFs, the level of regulation shown is for the highest TF in the hierarchy HilD > HilC > RtsA > InvF > RtsB > SprB.

### Identification of direct regulatory targets of SPI-1-associated TFs without genome sequence or annotation.

The following procedure was applied for each of HilD, HilC, RtsA, InvF, SprB, and RtsB. We used Rockhopper to analyze RNA-seq data without a reference genome sequence or annotation (version 2.02; default parameters were used except for omission of a reference genome) (61). We then selected any transcript fragments (“transfrags”) (62) whose RNA level was significantly different (*q* = <0.01) by >2-fold in cells lacking the TF and cells in which the TF was transiently expressed. We aligned these RNA sequences to the 14028s genome using CLC Genomics Workbench (version 6; default parameters). We then compared the positions of the starts of these regulated transfrags to the positions of ChIP-seq peaks for the corresponding TF. Significantly regulated transfrags were classified as direct regulatory targets if the 5′ end of the transfrag was determined to be ≤200 bp downstream or ≤100 bp upstream of a ChIP-seq peak.

### Analysis of overlapping HilD, HilC, and RtsA binding sites.

We selected all HilD or RtsA ChIP-seq peaks that were located within 100 bp of a HilC ChIP-seq peak. If multiple HilC peaks were found within 100 bp of a HilD/RtsA peak, we selected the HilC peak with the highest Fold Above Threshold (FAT) score. For 400 bp surrounding each of the HilC peaks, we determined the sequence read coverage on each strand for each of the two replicate ChIP-seq datasets for HilC and for HilD/RtsA (four ChIP-seq peaks were common to all 3 TFs). We normalized the sequence read coverage in each case to the highest value in the 400-bp window. We then combined data for both strands and for both replicates for each TF. These data are represented by the heat maps in Fig. 3B. Heat maps were generated using Java TreeView (contrast set to 3.0) (86).

### Hierarchical clustering analysis.

For all direct regulatory targets of SPI-1-associated TFs (see Table S3 in the supplemental material), as well as predicted direct targets of HilA, we identified annotated homologues in strain SL4/74 using BLAST (87) (the best match was selected unless no matches scored better than an *E* value of 1E^−10^). We then extracted expression profiles for these genes for the 29 RNA-seq experiments described in reference 4. We performed hierarchical clustering of the data using Cluster (version 3.0; “uncentered Pearson correlation,” “average linkage” settings) (88, 89). We then determined Pearson correlation coefficients for all pairwise comparisons of expression profiles. These correlation coefficients were displayed as a heat map using Java TreeView (86).

### rtPCR.

For analysis of *hilC* and *sprB*, 14028s cells were grown overnight in LB (0.17 M NaCl), subcultured 1 in 20 in high-salt LB (0.3 mM NaCl), and shaken at 50 rpm at 37°C to achieve an optical density at 600 nm (OD_600_) of 0.8 to 0.9, and arabinose was added (0.2% final concentration) for 7 min before cells were harvested. For analysis of *invF* 5′ UTR, RNA was taken from RNA-seq samples for CDS024 plus pBLP013. For analysis of *STM14_5569* 5′ UTR, RNA was taken from RNA-seq samples for CDS022 plus pBLP011. For analysis of *ybdQ*, *osmB*, and *galF* 5′ UTRs, RNA was taken from RNA-seq samples for CDS030 plus pCDS001. RNA was purified and treated with DNase I as previously described (83). RNA (1 to 2 µg) was reverse transcribed using Superscript III with 2 µl 100 µM random hexamer, following the manufacturer’s instructions. A control sample was treated identically but without addition of reverse transcriptase. Following reverse transcription, samples were diluted to 200 µl. cDNA was PCR amplified and analyzed by agarose gel electrophoresis. Oligonucleotides used for PCR are listed in Table S6 in the supplemental material.

### Accession numbers.

All raw RNA-seq and ChIP-seq data (.fastq file format) are available at the EBI ArrayExpress database with accession numbers E-MTAB-3987 and E-MTAB-3765.

## SUPPLEMENTAL MATERIAL

Figure S1  Genome coverage plots for SPI-1-associated TFs. Histograms show RNA or ChIP sequence read coverage across selected genomic regions. Genes shaded in red, blue, and black indicate positive regulation, negative regulation, and no regulation, respectively. Black arrowheads indicate the position of ChIP-seq peaks. (A) RNA-seq and ChIP-seq data for HilD, for the region encompassing *sinR*. (B) RNA-seq and ChIP-seq data for HilD, for the region encompassing *hilC* and *sprB*. (C) RNA-seq and ChIP-seq data for HilC, for the region encompassing *cspE*. (D and E) RNA-seq and ChIP-seq data for InvF, for the regions encompassing *sopE2* (D) and *STM14_1486* (*STM1239*) (E). (F) RNA-seq and ChIP-seq data for SprB, for the region encompassing *STM14_2227* (*STM1841*). (G) RNA-seq and ChIP-seq data for SprB, for the region encompassing *siiA* (first gene in SPI-4). (H) RNA-seq and ChIP-seq data for RtsB and HilD, for the region encompassing *flhDC*. (I) RNA-seq and ChIP-seq data for RtsB and HilD, for the region encompassing *stdA*. (J and K) RNA-seq data for HilA, for the regions encompassing *pspA* to *pspE* (J) and *yiaD* (K). (L) RNA-seq and ChIP-seq data for HilD, HilC, and RtsA, for the region encompassing *dapB*. DapZ, the sRNA that initiates within *dapB*, is indicated by brackets. Download Figure S1, PDF file, 0.4 MB

Figure S2  *hilC* and *sprB* are cotranscribed. An agarose gel shows products from rtPCR performed using primers that span the region between *hilC* and *sprB*. L, 100-bp ladder; +, PCR was performed using a sample that was generated with reverse transcriptase and RNA purified from cells transiently overexpressing HilD; −, PCR was performed using a control sample that was generated without adding reverse transcriptase and RNA purified from cells transiently overexpressing HilD (negative control); C, PCR was performed using a colony of *S*. Typhimurium (positive control). Download Figure S2, PDF file, 0.1 MB

Figure S3  Regulatory targets of InvF. Alignments of likely InvF-bound regions upstream of previously reported and novel InvF-bound regions are shown. The boxed region has been shown to be critical for InvF function at *sopB* and *sopE* (in strain SL1344) (46). Shaded bases are identical across all five regions. Arrowheads indicate the position of ChIP-seq peaks for InvF. Download Figure S3, PDF file, 0.1 MB

Figure S4  Identification of transfrags as 5′ UTRs using rtPCR. Agarose gels show products from rtPCR performed using primers that span the indicated regions. L, 100-bp ladder; +, PCR was performed using a sample that was generated with reverse transcriptase and RNA purified from cells transiently overexpressing HilD; −, PCR was performed using a control sample that was generated without adding reverse transcriptase and RNA purified from cells transiently overexpressing HilD (negative control); C, PCR was performed using a colony of *S*. Typhimurium (positive control). A black arrow to the right of each gel image indicates the expected PCR product size. Download Figure S4, PDF file, 0.1 MB

Table S1  Complete RNA-seq analysis for HilD, HilC, RtsA, InvF, SprB, RtsB, and HilA. Table S1, XLSX file, 2.8 MB

Table S2  List of all ChIP-seq peaks for HilD, HilC, RtsA, InvF, SprB, and RtsB. Table S2, XLSX file, 0.02 MB

Table S3  List of all direct regulatory targets for HilD, HilC, RtsA, InvF, SprB, and RtsB. Table S3, DOCX file, 0.03 MB

Table S4  List of transcript variants (5′ UTRs) regulated by SPI-1-associated TFs. Table S4, DOCX file, 0.01 MB

Table S5  Bacterial strains and plasmids used in this study. Table S5, DOCX file, 0.02 MB

Table S6  Oligonucleotides used in this study. Table S6, DOCX file, 0.02 MB
