# Supplementary figures and images for: Mapping the Regulatory Network for Salmonella enterica Serovar Typhimurium Invasion
Source: mBio. 2016 Sep 6;7(5):e01024-16. doi: 10.1128/mBio.01024-16 (PMC5013294; doi:10.1128/mBio.01024-16)

Figure S1

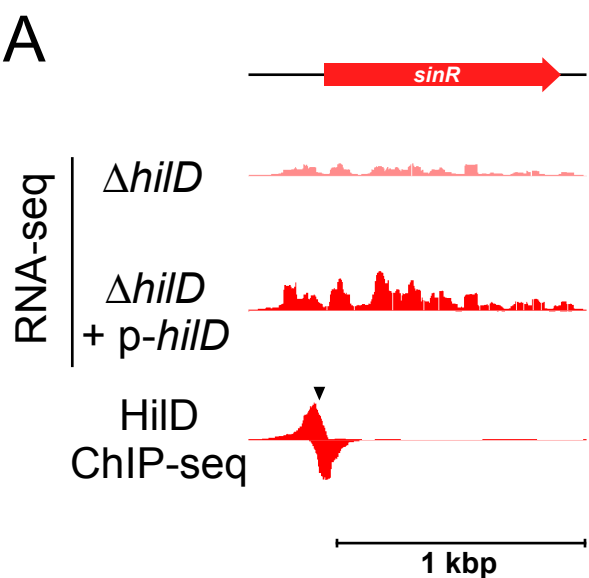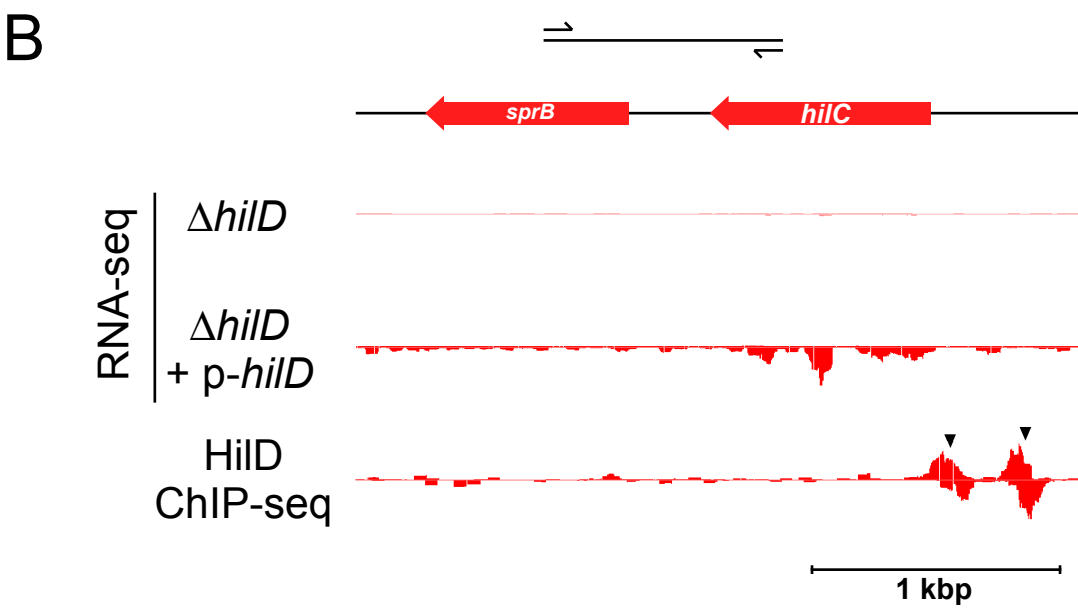

Figure S1

C

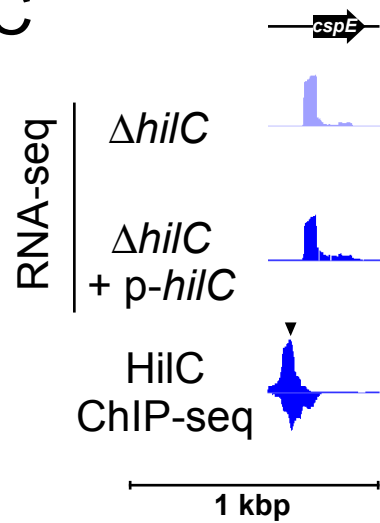

D

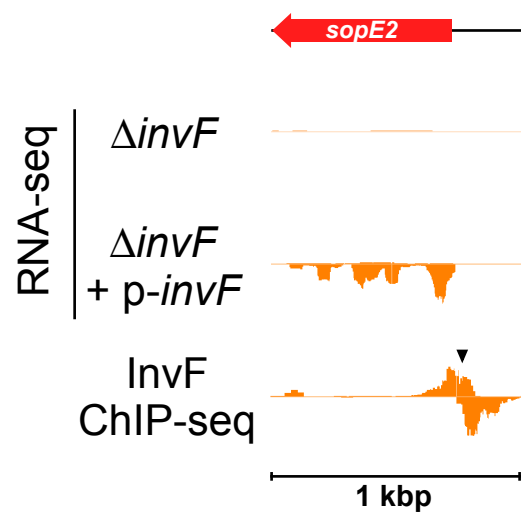

E

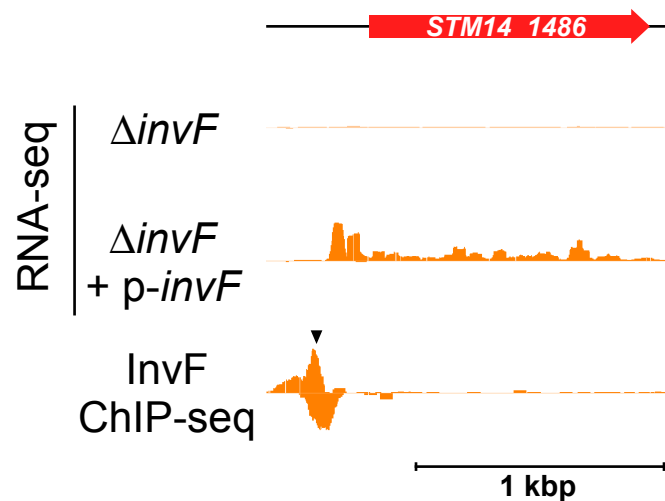

Figure S1

F

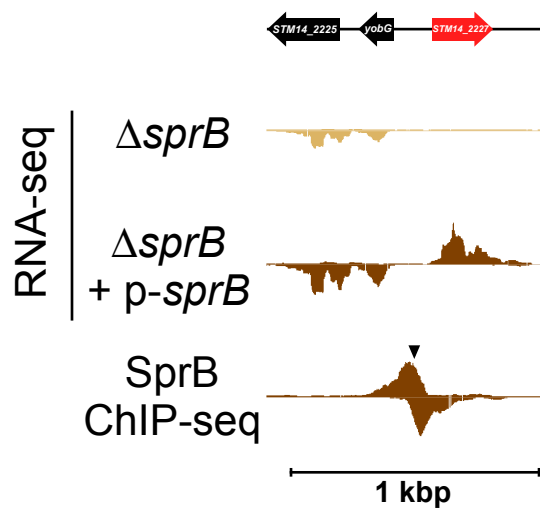

G

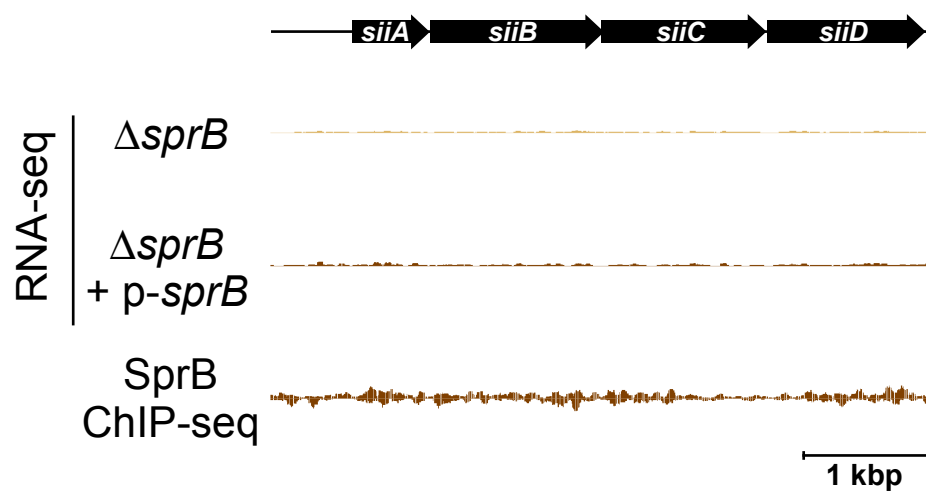

Figure S1

H

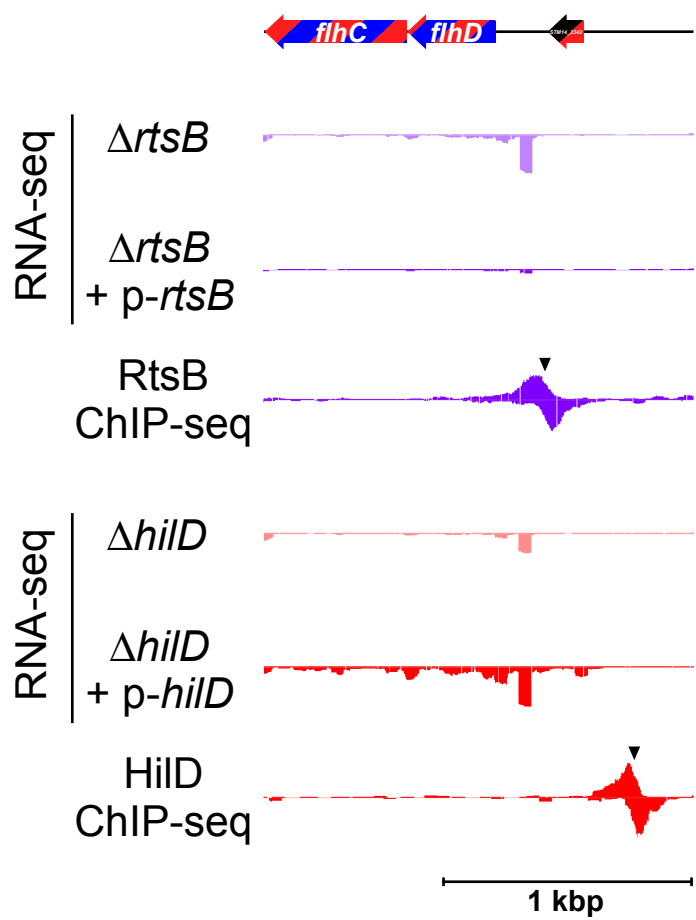

I

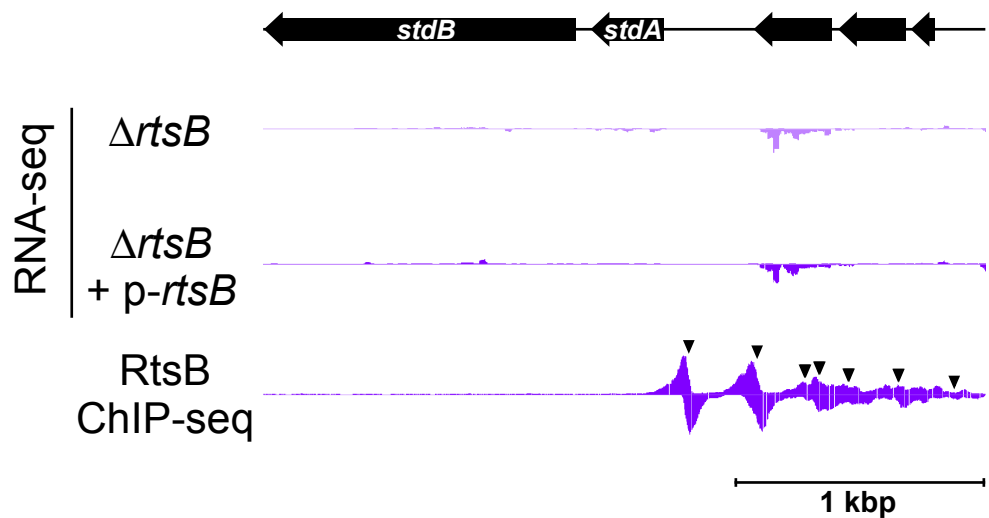

Figure S1

J

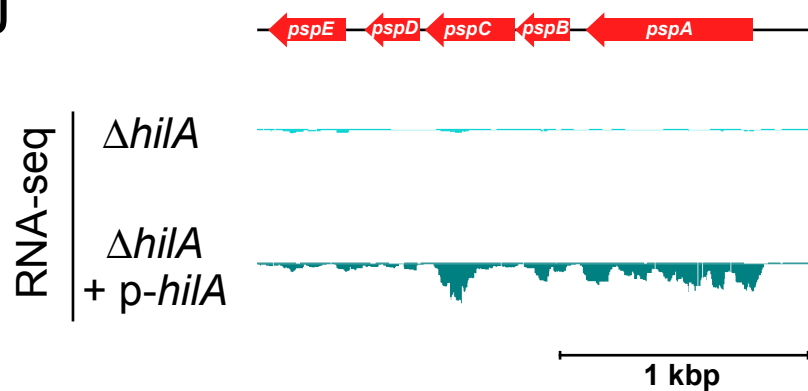

K

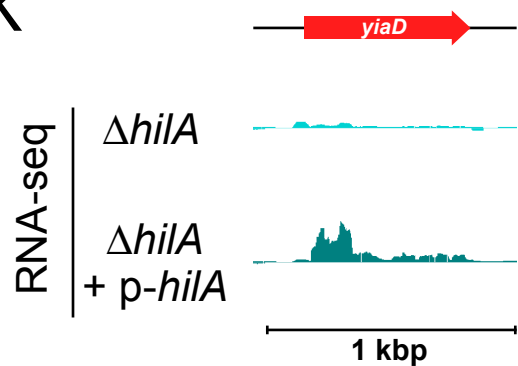

L

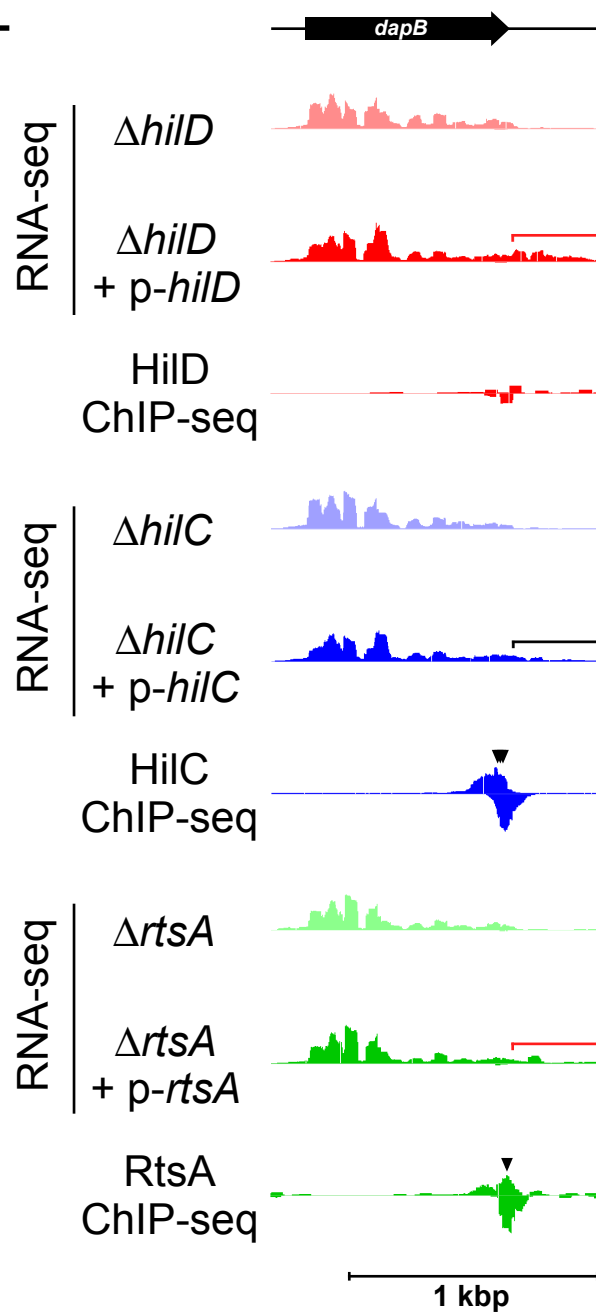

Supplement: Figure S1 — Genome coverage plots for SPI-1-associated TFs. Histograms show RNA or ChIP sequence read coverage across selected genomic regions. Genes shaded in red, blue, and black indicate positive regulation, negative regulation, and no regulation, respectively. Black arrowheads indicate the position of ChIP-seq peaks. (A) RNA-seq and ChIP-seq data for HilD, for the region encompassing sinR. (B) RNA-seq and ChIP-seq data for HilD, for the region encompassing hilC and sprB. (C) RNA-seq and ChIP-seq data for HilC, for the region encompassing cspE. (D and E) RNA-seq and ChIP-seq data for InvF, for the regions encompassing sopE2 (D) and STM14_1486 (STM1239) (E). (F) RNA-seq and ChIP-seq data for SprB, for the region encompassing STM14_2227 (STM1841). (G) RNA-seq and ChIP-seq data for SprB, for the region encompassing siiA (first gene in SPI-4). (H) RNA-seq and ChIP-seq data for RtsB and HilD, for the region encompassing flhDC. (I) RNA-seq and ChIP-seq data for RtsB and HilD, for the region encompassing stdA. (J and K) RNA-seq data for HilA, for the regions encompassing pspA to pspE (J) and yiaD (K). (L) RNA-seq and ChIP-seq data for HilD, HilC, and RtsA, for the region encompassing dapB. DapZ, the sRNA that initiates within dapB, is indicated by brackets. Download [file mbo004162976sf1.pdf]

Figure S2

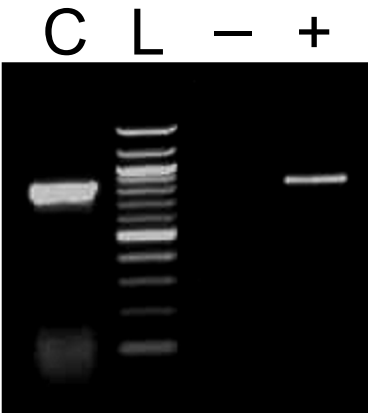

Supplement: Figure S2 — hilC and sprB are cotranscribed. An agarose gel shows products from rtPCR performed using primers that span the region between hilC and sprB. L, 100-bp ladder; +, PCR was performed using a sample that was generated with reverse transcriptase and RNA purified from cells transiently overexpressing HilD; −, PCR was performed using a control sample that was generated without adding reverse transcriptase and RNA purified from cells transiently overexpressing HilD (negative control); C, PCR was performed using a colony of S. Typhimurium (positive control). Download [file mbo004162976sf2.pdf]

# Figure S3

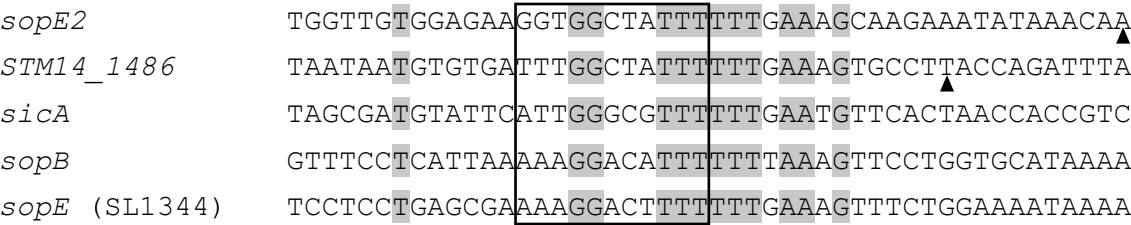

Supplement: Figure S3 — Regulatory targets of InvF. Alignments of likely InvF-bound regions upstream of previously reported and novel InvF-bound regions are shown. The boxed region has been shown to be critical for InvF function at sopB and sopE (in strain SL1344) (46). Shaded bases are identical across all five regions. Arrowheads indicate the position of ChIP-seq peaks for InvF. Download [file mbo004162976sf3.pdf]

Figure S4

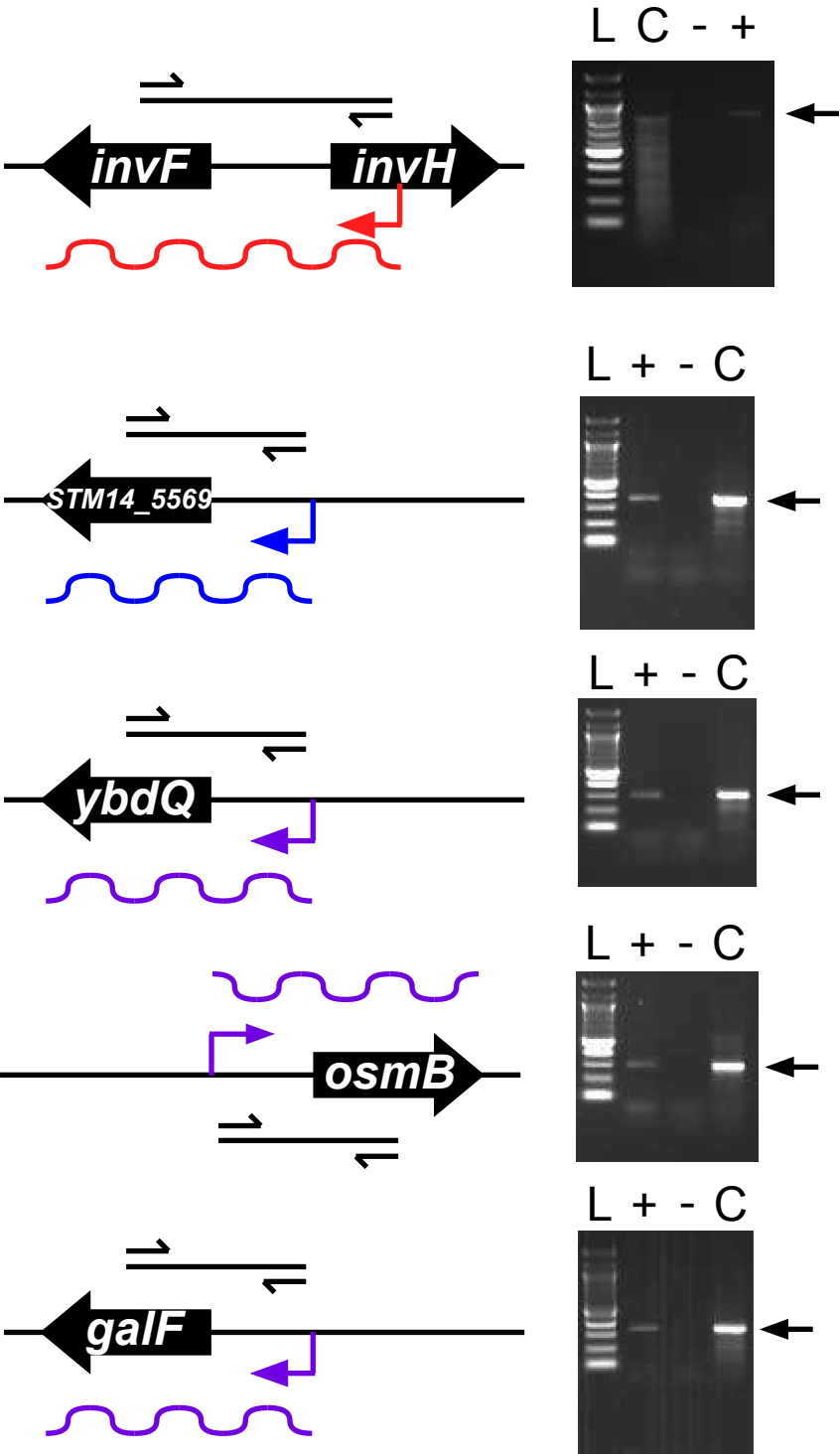

Supplement: Figure S4 — Identification of transfrags as 5′ UTRs using rtPCR. Agarose gels show products from rtPCR performed using primers that span the indicated regions. L, 100-bp ladder; +, PCR was performed using a sample that was generated with reverse transcriptase and RNA purified from cells transiently overexpressing HilD; −, PCR was performed using a control sample that was generated without adding reverse transcriptase and RNA purified from cells transiently overexpressing HilD (negative control); C, PCR was performed using a colony of S. Typhimurium (positive control). A black arrow to the right of each gel image indicates the expected PCR product size. Download [file mbo004162976sf4.pdf]
