# Supplementary material for: Mapping the Regulatory Network for Salmonella enterica Serovar Typhimurium Invasion
Source: mBio. 2016 Sep 6;7(5):e01024-16. doi: 10.1128/mBio.01024-16 (PMC5013294; doi:10.1128/mBio.01024-16)
Supplement: Table S3 — List of all direct regulatory targets for HilD, HilC, RtsA, InvF, SprB, and RtsB. [file mbo004162976st3.docx]

**Table S3. List of all direct regulatory targets for HilD, HilC, RtsA, InvF, SprB and RtsB.**

| **TF** | **Gene ID^a^** | **Common Name^a^** | **ST4/74 Name^b^** | **LT2 Name^c^** | **Fold Change**  **(log_2_)^d^** |
| --- | --- | --- | --- | --- | --- |
| HilD | *STM14_0358* | *sinR* | *SL0300* | *STM0304* | 1.30 |
| HilD | *STM14_1282* |  |  |  | 5.48 |
| HilD | *STM14_1283* |  |  | *STM05010* | 1.96 |
| HilD | *STM14_1612* | *lpxR* | *SL1263* | *STM1328* | 8.95 |
| HilD | *STM14_1613* |  | *SL1264* | *STM1329* | 5.17 |
| HilD | *STM14_1614* |  | *SL1265* | *STM1330* | 3.28 |
| HilD | *STM14_2340* | *flhC* | *SL1859* | *STM1924.S* | 2.16 |
| HilD | *STM14_2341* | *flhD* | *SL1860* | *STM1925* | 2.50 |
| HilD | *STM14_2342* |  |  |  | 5.19 |
| HilD | *STM14_3463* | *sprB* | *SL2846* | *STM2866* | 4.94 |
| HilD | *STM14_3465* | *hilC* | *SL2847* | *STM2867* | 3.48 |
| HilD | *STM14_3466* |  |  |  | 5.52 |
| HilD | *STM14_3467* | *orgC* | *SL2848* | *STM2868* | 3.18 |
| HilD | *STM14_3468* | *orgB* | *SL2849* | *STM2869* | 3.26 |
| HilD | *STM14_3469* | *orgA* | *SL2850* | *STM2870* | 5.31 |
| HilD | *STM14_3470* | *prgK* | *SL2851* | *STM2871* | 6.89 |
| HilD | *STM14_3471* | *prgJ* | *SL2852* | *STM2872* | 7.72 |
| HilD | *STM14_3472* | *prgI* | *SL2853* | *STM2873* | 7.83 |
| HilD | *STM14_3473* | *prgH* | *SL2854* | *STM2874* | 8.49 |
| HilD | *STM14_3474* | *hilD* | *SL2855* | *STM2875* | n/a* |
| HilD | *STM14_3475* | *hilA* | *SL2856* | *STM2876* | 8.79 |
| HilD | *STM14_3476* | *iagB* | *SL2857* | *STM2877* | 7.64 |
| HilD | *STM14_3486* | *spaS* | *SL2866* | *STM2887* | 6.09 |
| HilD | *STM14_3487* | *spaR* | *SL2867* | *STM2888* | 6.67 |
| HilD | *STM14_3488* | *spaQ* | *SL2868* | *STM2889* | 6.21 |
| HilD | *STM14_3489* | *spaP* | *SL2869* | *STM2890* | 6.82 |
| HilD | *STM14_3490* | *spaO* | *SL2870* | *STM2891* | 6.70 |
| HilD | *STM14_3491* | *invJ* | *SL2871* | *STM2892* | 6.99 |
| HilD | *STM14_3492* | *invI* | *SL2872* | *STM2893* | 6.95 |
| HilD | *STM14_3493* | *invC* | *SL2873* | *STM2894* | 6.95 |
| HilD | *STM14_3494* | *invB* | *SL2874* | *STM2895* | 6.15 |
| HilD | *STM14_3495* | *invA* | *SL2875* | *STM2896* | 6.47 |
| HilD | *STM14_3496* | *invE* | *SL2876* | *STM2897* | 6.86 |
| HilD | *STM14_3497* | *invG* | *SL2877* | *STM2898* | 7.19 |
| HilD | *STM14_3498* | *invF* | *SL2878* | *STM2899* | 8.92 |
| HilD | *STM14_3499* | *invH* | *SL2879* | *STM2900* | 6.56 |
| HilD | *STM14_3893* | *mcpC* | *SL3189* | *STM3216* | 1.91 |
| HilD | *STM14_5117* | *siiA* | *SL4193* | *STM4257* | n/a* |
| HilD | *STM14_5118* | *siiB* | *SL4194* | *STM4258* | 10.05 |
| HilD | *STM14_5119* | *siiC* | *SL4195* | *STM4259* | 9.38 |
| HilD | *STM14_5120* | *siiD* | *SL4196* | *STM4260* | 9.67 |
| HilD | *STM14_5121* | *siiE* | *SL4197* | *STM4261* | 2.48 |
| HilD | *STM14_5184* |  | *SL4247* | *STM4310* | 5.75 |
| HilD | *STM14_5185* |  | *SL4248* | *STM4312* | 5.51 |
| HilD | *STM14_5186* |  | *SL4249* | *STM4313* | 6.76 |
| HilD | *STM14_5187* | *rtsB* | *SL4250* | *STM4314* | 8.24 |
| HilD | *STM14_5188* | *rtsA* | *SL4251* | *STM4315* | 8.82 |
| HilD | *STM14_5189* |  |  |  | 3.61 |
| HilD | *STM14_5292* | *ytfK* | *SL4339* | *STM4406.S* | 3.45 |
| HilD | n/a | *mgrR* |  |  | 2.12 |
| HilD | n/a | ncRNA antisense to *mgrR* |  |  | 3.74 |
| HilD | n/a | ncRNA antisense to *hilA* 5' UTR |  |  | 4.23 |
| HilD | n/a | *invR* |  |  | 1.07 |
| HilC | *STM14_0048* | *nhaA* | *SL0040* | *STM0039* | 2.75 |
| HilC | *STM14_0049* | *nhaR* | *SL0041* | *STM0040* | 2.97 |
| HilC | *STM14_0219* | *yadB* | *SL0186* | *STM0185* | 1.65 |
| HilC | *STM14_0220* | *dksA* | *SL0187* | *STM0186* | 1.39 |
| HilC | *STM14_1612* | *lpxR* | *SL1263* | *STM1328* | 1.24 |
| HilC | *STM14_1702* | *ssaG* | *SL1340* | *STM1406* | 2.10 |
| HilC | *STM14_1705* | *ssaJ* | *SL1343* | *STM1409* | 1.02 |
| HilC | *STM14_2342* |  |  |  | 2.26 |
| HilC | *STM14_3463* | *sprB* | *SL2846* | *STM2866* | 1.20 |
| HilC | *STM14_3470* | *prgK* | *SL2851* | *STM2871* | 1.10 |
| HilC | *STM14_3471* | *prgJ* | *SL2852* | *STM2872* | 1.27 |
| HilC | *STM14_3473* | *prgH* | *SL2854* | *STM2874* | 1.15 |
| HilC | *STM14_3474* | *hilD* | *SL2855* | *STM2875* | 1.67 |
| HilC | *STM14_3475* | *hilA* | *SL2856* | *STM2876* | 5.41 |
| HilC | *STM14_3476* | *iagB* | *SL2857* | *STM2877* | 4.71 |
| HilC | *STM14_3495* | *invA* | *SL2875* | *STM2896* | 1.20 |
| HilC | *STM14_3496* | *invE* | *SL2876* | *STM2897* | 1.62 |
| HilC | *STM14_3497* | *invG* | *SL2877* | *STM2898* | 1.57 |
| HilC | *STM14_3498* | *invF* | *SL2878* | *STM2899* | 1.77 |
| HilC | *STM14_3499* | *invH* | *SL2879* | *STM2900* | 3.47 |
| HilC | *STM14_5184* |  | *SL4247* | *STM4310* | 2.89 |
| HilC | n/a | ncRNA overlaps *STM14_1614* | *SL1265* |  | 0.88 |
| HilC | *STM14_5569 5' UTR* |  | *SLp1098* | *PSLT046* | -0.30 |
| HilC | n/a | *invR* |  |  | 3.99 |
| HilC | n/a | ncRNA antisense to *STM14_5565* |  |  | 2.15 |
| RtsA | *STM14_0219* | *yadB* | *SL0186* | *STM0185* | 1.64 |
| RtsA | *STM14_0220* | *dksA* | *SL0187* | *STM0186* | 1.01 |
| RtsA | *STM14_1612* | *lpxR* | *SL1263* | *STM1328* | 4.87 |
| RtsA | *STM14_1614* |  | *SL1265* | *STM1330* | 2.28 |
| RtsA | *STM14_3467* | *orgC* | *SL2848* | *STM2868* | 1.35 |
| RtsA | *STM14_3468* | *orgB* | *SL2849* | *STM2869* | 1.50 |
| RtsA | *STM14_3469* | *orgA* | *SL2850* | *STM2870* | 1.73 |
| RtsA | *STM14_3470* | *prgK* | *SL2851* | *STM2871* | 2.04 |
| RtsA | *STM14_3471* | *prgJ* | *SL2852* | *STM2872* | 2.03 |
| RtsA | *STM14_3472* | *prgI* | *SL2853* | *STM2873* | 1.90 |
| RtsA | *STM14_3473* | *prgH* | *SL2854* | *STM2874* | 2.16 |
| RtsA | *STM14_3474* | *hilD* | *SL2855* | *STM2875* | 4.36 |
| RtsA | *STM14_3486* | *spaS* | *SL2866* | *STM2887* | 1.83 |
| RtsA | *STM14_3487* | *spaR* | *SL2867* | *STM2888* | 2.22 |
| RtsA | *STM14_3488* | *spaQ* | *SL2868* | *STM2889* | 2.57 |
| RtsA | *STM14_3489* | *spaP* | *SL2869* | *STM2890* | 2.00 |
| RtsA | *STM14_3490* | *spaO* | *SL2870* | *STM2891* | 2.19 |
| RtsA | *STM14_3491* | *invJ* | *SL2871* | *STM2892* | 2.24 |
| RtsA | *STM14_3492* | *invI* | *SL2872* | *STM2893* | 2.16 |
| RtsA | *STM14_3493* | *invC* | *SL2873* | *STM2894* | 2.56 |
| RtsA | *STM14_3494* | *invB* | *SL2874* | *STM2895* | 2.19 |
| RtsA | *STM14_3495* | *invA* | *SL2875* | *STM2896* | 2.69 |
| RtsA | *STM14_3496* | *invE* | *SL2876* | *STM2897* | 3.04 |
| RtsA | *STM14_3497* | *invG* | *SL2877* | *STM2898* | 3.10 |
| RtsA | *STM14_3498* | *invF* | *SL2878* | *STM2899* | 2.9 |
| RtsA | *STM14_3499* | *invH* | *SL2879* | *STM2900* | 3.84 |
| RtsA | *STM14_5184* |  | *SL4247* | *STM4310* | 4.78 |
| RtsA | *STM14_5546* | *repA2* |  | *PSLT023* | 1.20 |
| RtsA | n/a | *dapZ* |  |  | 2.66 |
| RtsA | n/a | ncRNA antisense to *slrP* |  |  | 1.25 |
| RtsA | n/a | ncRNA antisense to *mgrR* |  |  | 2.05 |
| RtsA | n/a | ncRNA within *dacB* |  |  | 2.54 |
| InvF | *STM14_1486* |  | *SL1177* | *STM1239* | 4.44 |
| InvF | *STM14_2244* | *sopE2* | *SL1784* | *STM1855* | 7.87 |
| RtsB | *STM14_0687* | *fepE* | *SL0577* | *STM0589* | -2.8 |
| RtsB | *STM14_0793* | *nagD* | *SL0663* | *STM0681* | -1.49 |
| RtsB | *STM14_1341* | *flgN* | *SL1108* | *STM1171* | -1.25 |
| RtsB | *STM14_1342* | *flgM* | *SL1109* | *STM1172* | -1.41 |
| RtsB | *STM14_1833* |  |  |  | 2.51 |
| RtsB | *STM14_1887* | *yddX* | *SL1494* | *STM1564* | -2.96 |
| RtsB | *STM14_2340* | *flhC* | *SL1859* | *STM1924.S* | -1.53 |
| RtsB | *STM14_2341* | *flhD* | *SL1860* | *STM1925* | -1.67 |
| RtsB | *STM14_2350* | *ftnB* | *SL1865* | *STM1932* | -1.72 |
| RtsB | *STM14_0998* | *bssR* | *SL0829* | *STM0853* | -2.19 |
| RtsB | *STM14_0713 5' UTR* | *ybdQ 5' UTR* | *SL0602* | *STM0614* | 0.26 |
| RtsB | *STM14_2062 5' UTR* | *osmB 5' UTR* | *SL1637* | *STM1705* | -0.71 |
| RtsB | *STM14_2592 5' UTR* | *galF 5' UTR* | *SL2075* | *STM2098* | 0.32 |
| SprB | *STM14_0398* |  | *SL0336* | *STM0341* | 2.60 |
| SprB | *STM14_0399* |  | *SL0337* | *STM0342* | 2.12 |
| SprB | *STM14_1560* | *mipA* | *SL1221* | *STM1286* | 2.66 |
| SprB | *STM14_1938* | *ugtL* | *SL1531* | *STM1601* | 2.95 |
| SprB | *STM14_1939* |  |  |  | 2.89 |
| SprB | *STM14_1940* | *sifB* | *SL1532* | *STM1602* | 3.46 |
| SprB | *STM14_2227* |  | *SL1770* | *STM1841* | 5.67 |
| SprB | *STM14_2573* | *wzzB* | *SL2056* | *STM2079* | 1.80 |
| SprB | *STM14_2953* | *ddg/lpxP* | *SL2369* | *STM2401* | 1.76 |
| SprB | *STM14_3069* |  |  |  | 2.19 |
| SprB | *STM14_3628* |  |  |  | 2.25 |
| SprB | *STM14_3629* | *ygdQ* | *SL2984* | *STM3006* | 2.17 |
| SprB | *STM14_3799* |  | *SL3112* | *STM3138* | 2.89 |
| SprB | *STM14_4212* | *yhgE* | *SL3466* | *STM3499* | 1.18 |
| SprB | *STM14_4214* |  |  |  | 1.25 |
| SprB | *STM14_4215* | *pckA* | *SL3467* | *STM3500* | 1.18 |
| SprB | *STM14_4465* | *yibP* | *SL3671* | *STM3705* | 1.12 |
| SprB | *STM14_4592* | *yidF* | *SL3771* | *STM3803* | 2.48 |
| SprB | *STM14_5096* |  |  | *STM4239* | 1.01 |
| SprB | *STM14_5097* | *yjbJ* | *SL4176* | *STM4240* | 1.32 |
| SprB | *STM14_5495* |  | *SL4502* | *STM4575* | 1.72 |
| SprB | *STM14_0927* |  |  |  | 2.81 |
| SprB | *STM14_0928* | *slrP* | *SL0776* | *STM0800* | 3.35 |
| SprB | n/a | *STnc520* |  |  | 4.08 |

^a^ Underlined gene names indicate known invasion genes. Double-underlined gene names indicate genes with highly correlated gene expression profiles with the known invasion genes (see Figure 7).

^b^ Gene name for homologue in strain ST4/74. Some genes in 14028s do not have an annotated homologue in ST4/74.

^c^ Gene name for homologue in strain LT2. Some genes in 14028s do not have an annotated homologue in LT2.

^d^ Fold change in RNA levels between cells expressing the corresponding TF and cells deleted for the TF-encoding gene. Asterisks indicate significantly regulated genes for which fold change numbers could not be determined. In the case of *siiA*, there was zero expression in the absence of HilD. In the case of *hilD*, we could not measure expression in the Δ*hilD* strain, but we know from other studies that HilD is autoregulated, consistent with the binding site for HilD upstream of the gene.
