# Supplementary material for: Mapping the Regulatory Network for Salmonella enterica Serovar Typhimurium Invasion
Source: mBio. 2016 Sep 6;7(5):e01024-16. doi: 10.1128/mBio.01024-16 (PMC5013294; doi:10.1128/mBio.01024-16)
Supplement: Table S4 — List of transcript variants (5′ UTRs) regulated by SPI-1-associated TFs. [file mbo004162976st4.docx]

**Table S4. List of transcript variants (5’ UTRs) regulated by SPI-1-associated TFs.**

| **Start** ^a^ | **Stop** ^a^ | **Strand** | **Gene^b^** | **LT2 gene name^c^** | **TF Regulator(s)** | **Fold Change^d^** |
| --- | --- | --- | --- | --- | --- | --- |
| 38400* | 38315* | - | *STM14_5569* | *PSLT046* | HilC | 3.22 |
| 678374 | 678310 | - | *ybdQ* | *STM0614* | RtsB | -1.62 |
| 1810471 | 1810593 | + | *osmB* | *STM1705* | RtsB | -1.17 |
| 2231527 | 2231429 | - | *galF* | *STM2098* | RtsB | -1.56 |

^a^ predicted start/stop genome coordinates for a given 5’ UTR. Numbers marked with an asterisk indicate a transcript encoded on the virulence plasmid.

^b^ Gene with which a regulated 5’ UTR is associated.

^c^ Gene name for homologue in strain LT2.

^d^ Fold change (log_2_) in RNA level for cells overexpression the corresponding TF versus cells in which the TF-encoding gene was deleted.
