# Supplementary material for: Mapping the Regulatory Network for Salmonella enterica Serovar Typhimurium Invasion
Source: mBio. 2016 Sep 6;7(5):e01024-16. doi: 10.1128/mBio.01024-16 (PMC5013294; doi:10.1128/mBio.01024-16)
Supplement: Table S5 — Bacterial strains and plasmids used in this study. [file mbo004162976st5.docx]

**Table S5. Bacterial strains and plasmids used in this study.**

| ***S.* Typhimurium Strains** | **Description** | **Source** |
| --- | --- | --- |
| 14028s | Wild-type | (1) |
| AMD475 | 14028s *hilD*-FLAG_3_ | This study |
| AMD474 | 14028s *hilC*-FLAG_3_ | This study |
| AMD476 | 14028s *rtsA*-FLAG_3_ | This study |
| AMD478 | 14028s *invF*-FLAG_3_ | This study |
| AMD508 | 14028s *sprB*-FLAG_3_ | This study |
| AMD477 | 14028s *rtsB*-FLAG_3_ | This study |
| AMD473 | 14028s *hilA*-FLAG_3_ | This study |
| CDS024 | 14028s Δ*hilD*::*kan^R^* | This study |
| CDS022 | 14028s Δ*hilC*::*kan^R^* | This study |
| CDS020 | 14028s Δ*rtsA*::*kan^R^* | This study |
| CDS028 | 14028s Δ*invF*::*kan^R^* | This study |
| CDS026 | 14028s Δ*sprB*::*kan^R^* | This study |
| CDS030 | 14028s Δ*rtsB*::*kan^R^* | This study |
| CDS032 | 14028s Δ*hilA*::*kan^R^* | This study |
|  |  |  |
| **Plasmids** |  |  |
| pBAD24 | pBAD24 (empty vector) | (2) |
| pBLP013 | pBAD24-*hilD* | This study |
| pBLP011 | pBAD24-*hilC* | This study |
| pBLP010 | pBAD24-*rtsA* | This study |
| pCDS003 | pBAD24-*invF* | This study |
| pCDS002 | pBAD24-*sprB* | This study |
| pCDS001 | pBAD24-*rtsB* | This study |
| pCDS004 | pBAD24-*hilA* | This study |
