# Supplementary material for: Mapping the Regulatory Network for Salmonella enterica Serovar Typhimurium Invasion
Source: mBio. 2016 Sep 6;7(5):e01024-16. doi: 10.1128/mBio.01024-16 (PMC5013294; doi:10.1128/mBio.01024-16)
Supplement: Table S6 — Oligonucleotides used in this study. [file mbo004162976st6.docx]

**Table S6. Oligonucleotides used in this study.**

| **Oligonucleotide** | **Sequence** | **Application** |
| --- | --- | --- |
| JW3983 | GTACCTGACGATAATTGCGCGTTACCGATTGCGGTAGGAATGAATGGCGGTGGCGACTAC | FLAG-tagging *sprB* (3) |
| JW3984 | AAGCTCAACACATGGTCATAAATGATAAAAAATATTTTAGTTGGGAGCTCACTACTTGTC | FLAG-tagging *sprB* (3) |
| JW3987 | CATTTTAAGATTACACCGCTTAGCTTTATGCGTACAATGAACCATGGCGGTGGCGACTAC | FLAG-tagging *hilC* (3) |
| JW3988 | GCAAACAGATAGTAACGTTTAAAATAATTTCACAAATCAGTTGGGAGCTCACTACTTGTC | FLAG-tagging *hilC* (3) |
| JW3991 | TATTTTAAAACTACGCCATCGACATTCATAAAAATGGCGAACCATGGCGGTGGCGACTAC | FLAG-tagging *hilD* (3) |
| JW3992 | TAAAAATCTTTACTTAAGTGACAGATACAAAAAATGTTAGTTGGGAGCTCACTACTTGTC | FLAG-tagging *hilD* (3) |
| JW3995 | TGGTTCAAAAGATGGAAACAGGATCCCCGCTTGATTAAATTACGGGGCGGTGGCGACTAC | FLAG-tagging *hilA* (3) |
| JW3996 | TGATAAAAAAATAATGCATATCTCCTCTCTCAGATTTTAGTTGGGAGCTCACTACTTGTC | FLAG-tagging *hilA* (3) |
| JW3999 | GTTTCGCCGCGGAAATTATCAAATATTATTCAATTGGCAGACAAAGGCGGTGGCGACTAC | FLAG-tagging *invF* (3) |
| JW4000 | CACATGCCAGCACTCTGGCCAAAAGAATATGTGTCTTCAGTTGGGAGCTCACTACTTGTC | FLAG-tagging *invF* (3) |
| JW4075 | ATCCATTTGATGCTGAATGAGTTTTACATATCAGTCGATATTACGGGCGGTGGCGACTAC | FLAG-tagging *rtsB* (3) |
| JW4076 | GCCTTGCCTACCACTCTACCAACATTTTAGGAAAAATTAGTTGGGAGCTCACTACTTGTC | FLAG-tagging *rtsB* (3) |
| JW4079 | GAATTTTTAGCGTTTTTATCTTCCTCTCGTCATCAATATGTTAATGGCGGTGGCGACTAC | FLAG-tagging *rtsA* (3) |
| JW4080 | GTTTTGCTTTGTTCTTATACTGCATTGTCAGATATCTCAGTTGGGAGCTCACTACTTGTC | FLAG-tagging *rtsA* (3) |
| JW4788 | GAGGAATTCACCATGCCACATTTTAATCCTGTTCC | pCDS004 cloning |
| JW4789 | TCCCCGGGTACCATGTTACCGTAATTTAATCAAGCG | pCDS004 cloning |
| JW4790 | GAGGAATTCACCATGAGAAATGTAATTATATACGGTATTAACTGG | pCDS002 cloning |
| JW4791 | TCCCCGGGTACCATGTTAATTCATTCCTACCGCAAT | pCDS002 cloning |
| JW4792 | GAGGAATTCACCATGCTAAATACGCAGGAAGTACTTAAAGAAG | pCDS003 cloning |
| JW4793 | TCCCCGGGTACCATGTCATTTGTCTGCCAATTGAAT | pCDS003 cloning |
| JW4794 | GAGGAATTCACCATGAGATATCTGACAATGCAGTATAAGAAC | pCDS001 cloning |
| JW4795 | TCCCCGGGTACCATGTTACGTAATATCGACTGATATGTAAAACT | pCDS001 cloning |
| JW5161 | CCGCTGAAGAGGTGAGTT | *hilC*-*sprB* rtPCR |
| JW5162 | CGATCTGCCACTACCAAA | *hilC*-*sprB* rtPCR |
| JW6699 | TGCCAGACAAATAGCAGA | *ybdQ* 5' UTR rtPCR |
| JW6701 | ACCATCGTTTGCAGACG | *ybdQ* 5' UTR rtPCR |
| JW6702 | AGTGACTATCACCTGCG | *osmB* 5' UTR rtPCR |
| JW6704 | TAATGTACTGCCGTCCG | *osmB* 5' UTR rtPCR |
| JW6705 | GAGCGTGCCATCATTATC | *galF* 5' UTR rtPCR |
| JW6707 | CTCAAGAAGTGATTCAAGTTC | *galF* 5' UTR rtPCR |
| JW6708 | TCGGTCATGAGTTGCTCT | *invF* 5' UTR rtPCR |
| JW6709 | CAGAAAATGACATATGCAGC | *invF* 5' UTR rtPCR |
| JW6710 | CCAGTTCGACTGGTCTCA | *STM14_5569* 5' UTR rtPCR |
| JW6712 | CCAGGTAATCATGTTTAGC | *STM14_5569* 5' UTR rtPCR |

1. Jarvik T, Smillie C, Groisman EA, & Ochman H (2010) *J. Bacteriol.* **192,** 560-567.

2. Guzman L-M, Belin D, Carson MJ, & Beckwith JR (1995) *J. Bacteriol.* **177,** 4121-4130.

3. Stringer AM, Singh N, Yermakova A, Petrone BL, Amarasinghe JJ, Reyes-Diaz L, Mantis NJ, & Wade JT (2012) *PLoS One* **7,** e44841.
